# Probing the causes of thermal hysteresis using tunable *N*_agg_ micelles with linear and brush-like thermoresponsive coronas†
†Electronic supplementary information (ESI) available: NMR spectra of small molecules and polymers, SEC chromatograms of the polymers, DLS, SLS and turbidimetry data for the micelles, a discussion of the chain density of micelles **11–15**, additional calculations regarding the core composition of polymers **1–5** and definitions and calculations related to the light scattering data. See DOI: 10.1039/c6py01191h


**DOI:** 10.1039/c6py01191h

**Published:** 2016-08-09

**Authors:** L. D. Blackman, M. I. Gibson, R. K. O'Reilly

**Affiliations:** a Dept. of Chemistry , University of Warwick , Gibbet Hill Road , Coventry , CV4 7AL , UK . Email: r.k.o-reilly@warwick.ac.uk; b Warwick Medical School , University of Warwick , Gibbet Hill Road , Coventry , CV4 7AL , UK . Email: m.i.gibson@warwick.ac.uk

## Abstract

Self-assembled thermoresponsive polymers in aqueous solution have great potential as smart, switchable materials for use in biomedical applications. In recent years, attention has turned to the reversibility of these polymers’ thermal transitions, which has led to debate over what factors influence discrepancies in the transition temperature when heating the system compared to the temperature obtained when cooling the system, known as the thermal hysteresis. Herein, we synthesize micelles with tunable aggregation numbers (*N*_agg_) whose cores contain poly(*n*-butyl acrylate-*co-N*,*N*-dimethylacrylamide) (p(*n*BA-*co*-DMA)) and four different thermoresponsive corona blocks, namely poly(*N*-isopropylacrylamide) (pNIPAM), poly(*N*,*N*-diethylacrylamide) (pDEAm), poly(diethylene glycol monomethyl ether methacrylate) (pDEGMA) and poly(oligo(ethylene glycol) monomethyl ether methacrylate) (pOEGMA). By studying their thermoresponsive behavior, we elucidate the effects of changing numerous important characteristics both in the thermoresponsive chain chemistry and architecture, and in the structure of their self-assemblies. Our findings demonstrate large deviations in the reversibility between the self-assemblies and the corresponding thermoresponsive homopolymers; specifically we find that micelles whose corona consist of polymers with a brush-like architecture (pDEGMA and pOEGMA) exhibit irreversible phase transitions at a critical chain density. These results lead to a deeper understanding of stimuli-responsive self-assemblies and demonstrate the potential of tunable *N*_agg_ micelles for uncovering structure–property relationships in responsive polymer systems.

## Abstract 2

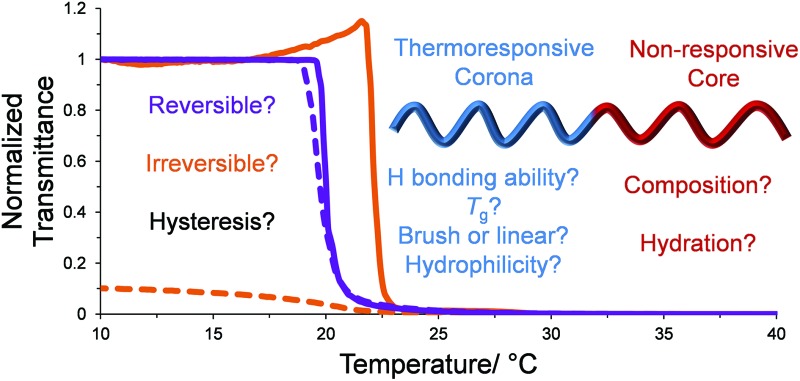
Self-assembled thermoresponsive polymers in aqueous solution have great potential as smart, switchable materials for use in biomedical applications.

## Introduction

The understanding and utilization of stimuli-responsive polymer materials has opened up new avenues in potential applications such as their use as drug delivery vehicles,^1–5^ smart surface coatings,^6,7^ for detection of specific molecules or ions,^6,8–11^ and for enhanced oil recovery^12^ to name just a few. These materials respond dramatically to subtle environmental changes such as light, pH, CO_2_, glucose or redox changes.^7,13–19^ One of the most studied and utilized stimuli in the literature is temperature as it offers a simple means of non-invasively altering the polymers’ environment.^14,19–21^ These thermoresponsive polymers exhibit a change in their solubility over a temperature range and reports of thermoresponsive behavior in both aqueous and organic solutions exist. The critical temperature at which this change in solubility occurs is known as an upper or lower critical solution temperature (UCST or LCST respectively).^19,21^ Above the UCST or below the LCST, the polymer and solvent exist as a single phase. Phase separation occurs if a polymer solution is heated above its LCST or cooled below its UCST and is normally observable by variable temperature turbidimetry, microcalorimetry, or NMR spectroscopy. This class of polymer materials has gathered interest in the medical field by virtue of the fact that they can exploit subtle temperature differences, for example between healthy or cancerous cells, for diagnostic, therapeutic or theranostic purposes,^1,22,23^ or for the design of smart injectable hydrogels for tissue engineering.^24,25^ Of these thermoresponsive polymer systems, poly(*N*-isopropylacrylamide) (pNIPAM) in aqueous solution is one of the most widely studied.^20,26^ This is because the polymer exhibits an LCST close to body temperature and this transition temperature is relatively insensitive to other environmental conditions such as pH or salt concentration making it an attractive candidate for biological applications.^26^ Although an important polymer, of which new potential applications are still being realized, several limitations currently exist including its lack of biodegradability, and its slow reversibility in certain systems, known as a thermal hysteresis;^26^ the latter of which may limit its applicability as a fully reversible, switchable smart material. As such, new thermoresponsive homo- and copolymers have been sought with lower or negligible hysteresis such that the transition occurs at the same temperature regardless of whether the system is being heated or cooled.^27–33^ In studying new thermoresponsive polymers, a greater understanding of the factors surrounding thermal hysteresis has been developed. For instance, structurally it has been shown that the hydrogen bonding character of the polymer side chain has a marked effect on the reversibility.^34,35^ In the case of pNIPAM, it has been shown using multiple angle light scattering, ultrasensitive differential scanning calorimetry and FT-IR analysis that the polymer undergoes several distinct conformations in the globule-to-coil transition owing to intramolecular hydrogen bonding between the amide repeat units, which leads to irreversibility in this system.^34–36^ Similar analysis has been performed on a polymer analogue that cannot form polymer–polymer hydrogen bonding interactions, namely poly(*N*,*N*-diethylacrylamide) (pDEAm), which did not exhibit the same behavior.^37^ It has also been postulated that the glass transition temperature (*T*_g_) may play a role in the reversibility of thermoresponsive polymer systems.^21,38^ Evidence suggests that polymers that exhibit a *T*_g_ below their LCST show transitions with lower thermal hysteresis than those whose LCST is above the *T*_g_ because of the increased mobility of the chains, which can facilitate easier redissolution.^21,38^ Polymers whose transition temperature is below their *T*_g_ are partially vitrified in the globular state so exhibit much slower rehydration kinetics.^39^ A loose trend in increasing hysteresis with *T*_g_ was also observed by Seuring and Agarwal in UCST type polymers.^40^

The advent of “living” polymerization and reversible-deactivation radical polymerization (RDRP) techniques has given access to amphiphilic block copolymers.^41–43^ These form higher order self-assembled polymer structures, such as micelles, worms and vesicles, and are only synthetically possible because of the ability to covalently attach incompatible domains (blocks) that micro-phase separate both in the bulk and in a selective solvent for one of the domains.^44–46^ There has been great interest in the synthesis and utilization of self-assembled amphiphilic block copolymers that include stimuli-responsive polymers as one or more of the blocks, for instance the incorporation of thermoresponsive blocks.^47–54^ These can undergo reversible morphological transitions such as micelles to vesicles, vesicles to unimers and micelles to unimers.^49–54^ However, when using pNIPAM as the responsive block, it has been shown that these transitions occur very slowly,^53,55^ with micelle to vesicle transitions occurring in the order of days to several weeks. Further study revealed that using a core-forming block containing methyl acrylate instead of *tert*-butyl acrylate resulted in a faster micelle to vesicle transition by lowering the *T*_g_ of the core-forming block.^56^ Additionally, Jiang and Chen *et al.* have shown that the reversibility in this system could be further improved by using a core-forming alkyl end group^57^ as opposed to the hydrophobic polymer blocks used in the studies by our group. These micelles were only lightly associated, which facilitated the rearrangement of the pNIPAM block by increasing the overall mobility of the chains.

In recent years, attention has turned to the effect of subtle changes in the structure of self-assemblies on the thermoresponsive behavior. Wang and Li synthesized a series of *n*-dodecyl terminated pNIPAM polymers with various molecular weights.^58^ These were self-assembled into micelles in aqueous solution, whose cores comprised solely of the terminal alkyl chain. The aggregation number (*N*_agg_), defined as the number of polymer chains per micelle, was then altered by adding the anionic surfactant sodium *n*-dodecyl sulfate (SDS), which could associate to the end groups of the polymer chains, thereby lowering the average *N*_agg_. It was found that the addition of SDS to the micelles increased the thermal transition temperature by reducing the overall hydrophobicity of the polymer end groups. The effect of SDS increasing the cloud point of the pNIPAM chains was more pronounced in lower molecular weight pNIPAM chains because their transition temperatures were initially most affected by the presence of the hydrophobic end group.^58^ Zhang and coworkers looked at the effect of the corona conformation within ABA and AB block copolymers with hydrophobic poly(styrene) A blocks and thermoresponsive pNIPAM B blocks, which formed either flower-like micelles with looped pNIPAM coronas, or classical crew-cut micelles respectively.^59^ It was found that micelles with a looped corona topology had a lower transition temperature, which occurred over a smaller temperature range than those formed from the AB diblock copolymer, as assessed by turbidimetry and ^1^H NMR spectroscopy of the dilute solutions. This was a consequence of the differences in the loss of entropy associated with the collapse of the corona chains between each set of micelles.^59^ Both these examples show that simply the arrangement of the chains in the micellar structure plays a dominant role in determining the overall thermoresponsive behavior.

Recently, we reported the synthesis of micelles formed from AB diblock copolymers with a thermoresponsive pNIPAM corona-forming block and a statistical copolymer of hydrophilic *N*,*N*-dimethylacrylamide (DMA) and hydrophobic *n*-butyl acrylate (*n*BA) as the core-forming block.^60^ These micelles underwent macroscopic precipitation upon heating, in a similar fashion to thermoresponsive homopolymers, owing to the change in solubility of the pNIPAM corona. We found that micelles with a tunable *N*_agg_ could be obtained by varying the composition of hydrophobic *n*BA in the core-forming block. Although the transition temperatures across the series were independent of core composition or *N*_agg_, the degree of hysteresis was found to increase as a function of core hydrophobicity. It was postulated that these differences were in fact a result of differences in core hydration, another important factor which determines reversibility in thermoresponsive self-assemblies (*vide infra*).

**Fig. 1:**
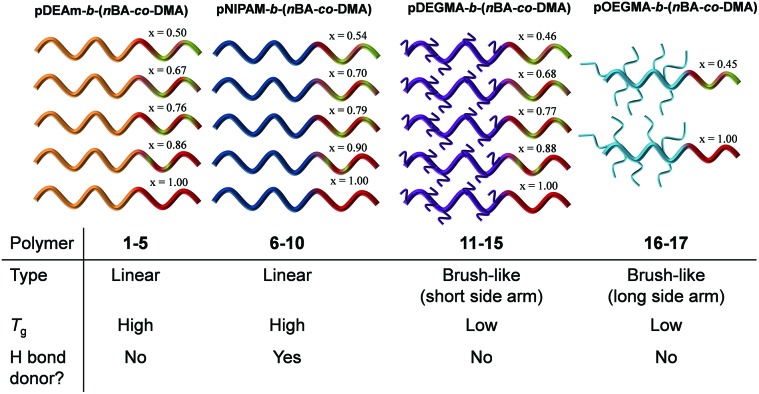
Illustration of the thermoresponsive diblock copolymers used in this study. Key: *x* = mol% *n*BA in the core-forming block. Below is a table outlining the differences in the corona blocks’ properties.

**Scheme 1:**
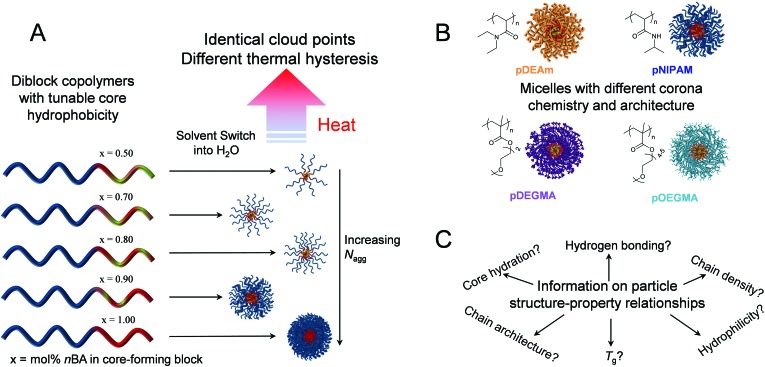
A: Schematic of one series of diblock copolymers with identical corona-forming blocks and tunable p(*n*BA-*co*-DMA) core compositions that self-assemble in water to yield micelles with a tunable *N*_agg_. The resulting particles show identical cloud points but different thermal hysteresis when heated in solution. B: Design of four micellar series with different corona-forming blocks with distinct chemistry and architecture, but whose cores contain the same p(*n*BA-*co*-DMA) compositions. In each case, the chemical structure of the corona block is shown. C: Studying the thermoresponsive behaviors of the four micellar series gives information on the structure–property relationships regarding thermal hysteresis in thermoresponsive self-assemblies.

**Scheme 2:**
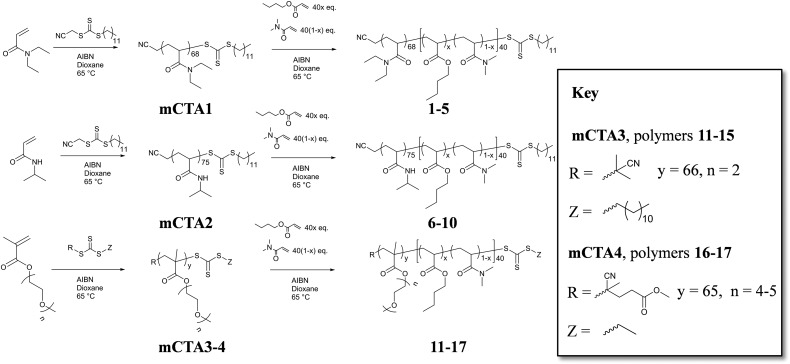
Synthesis of the four corona-forming macroCTA blocks (**mCTA1–4**) and their subsequent chain extension to yield the amphiphilic diblock copolymers (**1–17**).

Following on from these findings, herein we study the effects of micellar structure on the thermal hysteresis of four distinct thermoresponsive polymers, namely pNIPAM, pDEAm, poly(diethylene glycol monomethyl ether methacrylate) (pDEGMA) and poly(oligo(ethylene glycol) monomethyl ether methacrylate) (pOEGMA), the properties of which are outlined in Fig. 1. For each corona, a set of micelles with tunable *N*_agg_ was prepared using the previously studied p(*n*BA-*co*-DMA) core-forming block with varying core hydrophobicity (Schemes 1 and 2). The effect of corona hydrogen bonding ability, hydrophilicity, *T*_g_, core composition and *N*_agg_ of the micelles (which in turn relates to coronal chain confinement) on the thermoresponsive behavior of the micelles were studied. More generally, we also demonstrate that tunable *N*_agg_ micelles can be a powerful tool for uncovering structure–property relationships in stimuli-responsive self-assemblies (Scheme 1).

## Experimental

### Materials

The solvents petroleum ether (40–60 °C), diethyl ether, dichloromethane, ethyl acetate and acetone, and the reagents cyanomethyl dodecyl trithiocarbonate, 4,4′-azobis(4-cyanovaleric acid) (ACVA) and 4-dimethylaminopyridine (DMAP) were purchased from Sigma Aldrich and used as received. *N*-(3-Dimethylaminopropyl)-*N*′-ethylcarbodiimide hydrochloride (EDC·HCl) was purchased from Alpha Aesar and used as received. 1,4-Dioxane, and the monomers DEGMA, OEGMA (*M*_n_ = 300 g mol^–1^), *n*BA and DMA were purchased from Sigma Aldrich and passed through a column of basic alumina prior to use. NIPAM was purchased from Sigma Aldrich and recrystallized from a toluene–hexane mixture prior to use. 2,2′-Azobisisobutyronitrile (AIBN) was purchased from Sigma Aldrich and recrystallized from methanol prior to use. Dialysis membrane (MWCO = 3.5–5 kDa) was purchased from Spectra/Por. The synthesis of *N*,*N*-diethylacrylamide,^61^ 2-cyano-2-propyl dodecyl trithiocarbonate,^62^ 4-cyano-4-(((ethylthio)carbonothioyl)thio) pentanoic acid^63^ have been described previously in the literature.

### Small molecule and polymer analysis

SEC analyses of polymers **mCTA2** and **6–10** were performed on a Varian PL-GPC 50 Plus instrument fitted with mixed C columns and an RI detector using 5 mM NH_4_BF_4_ in *N*,*N*-dimethylformamide (DMF) as the eluent. SEC analyses of polymers **mCTA1**, **mCTA3**, **mCTA4**, **1–5** and **11–17** were performed on a Varian PL-GPC 50 Plus instrument fitted with mixed C columns and an RI detector using tetrahydrofuran (THF) containing 2% triethylamine (TEA) as the eluent. *M*_n_ values determined by SEC were calculated using poly(methyl methacrylate) standards. Additional triple detection SEC analysis was performed on **mCTA1** using 5 mM NH_4_BF_4_ in DMF as the eluent.

^1^H NMR spectroscopy was performed at 300 MHz on a Bruker Avance III HD-300 or a Bruker Avance AV-300 spectrometer, or at 400 MHz a Bruker Avance III HD-400 spectrometer. ^13^C NMR was performed at 75 MHz or 100 MHz on a Bruker Avance AV-300 or a Bruker Avance III HD-400 spectrometer respectively. For ^1^H and ^13^C NMR spectroscopy, chemical shifts (*δ*) in parts per million (ppm) are reported relative to the residual CHCl_3_ solvent peak at 7.26 ppm or 77.0 ppm, respectively.

High resolution electrospray ionization time of flight mass spectrometry (HRMS (ESI-ToF)) was performed on a Bruker MaXis mass spectrometer. Fourier transform infra-red (FT-IR) spectroscopy was performed on a Perkin-Elmer Spectrum 100 FT-IR spectrometer.

### Synthetic procedures

#### Preparation of methyl 4-cyano-4-(((ethylthio)carbonothioyl)thio) pentanoate

4-Cyano-4-(((ethylthio)carbonothioyl)thio) pentanoic acid (1.00 g, 3.8 mmol) was dissolved in methanol (50 mL) and EDC·HCl (1.46 g, 7.6 mmol) and DMAP (46 mg, 0.76 mmol) were added. After 18 h, a further equivalent of EDC·HCl (1.46 g, 7.6 mmol) and DMAP (46 mg, 0.76 mmol) were added and the reaction mixture was stirred for a further 18 h. The solvent was removed under reduced pressure and the crude product dissolved in dichloromethane (200 mL) and washed with water (3 × 300 mL) and brine (300 mL). The product was purified by column chromatography (SiO_2_ using 1 : 3 ethyl acetate : petroleum ether) to yield a yellow solid. Yield = 855 mg (81%). ^1^H NMR (300 MHz, CDCl_3_) *δ*/ppm: 3.70 (3H, s, COOC**H**_3_), 3.33 (2H, q, ^3^ *J*_H–H_ = 7.4 Hz, SC**H**_2_CH_3_), 2.65–2.57 (2H, m, C(CN)(CH_3_)CH_2_C**H**_2_), 2.56–2.35 (2H, m, C(CN)(CH_3_)C**H**_2_CH_2_), 1.87 (3H, s, C(CN)(C**H**_3_)CH_2_CH_2_), 1.35 (3H, t, ^3^ *J*_H–H_ = 7.4 Hz, SCH_2_C**H**_3_). ^13^C NMR (75 MHz, CDCl_3_) *δ*/ppm: 216.7 (**C** =S), 171.9 (**C** =O), 118.9 (C(**C**N)(CH_3_)CH_2_CH_2_), 52.1 (COO**C**H_3_), 46.3 (**C**(CN)(CH_3_)CH_2_CH_2_), 33.8 (S**C**H_2_CH_3_), 31.3 (C(CN)(CH_3_)CH_2_ **C**H_2_), 29.5 (C(CN)(CH_3_)**C**H_2_CH_2_), 24.8 (C(CN)(**C**H_3_)CH_2_CH_2_), 12.7 (SCH_2_ **C**H_3_). FT-IR (neat) *ν*/cm^–1^: 2965, 2934, 2848 (C–H stretch), 2237 (C≡N stretch), 1737 (C=O stretch). HRMS (ESI-TOF) *m*/*z*: [M + Na]^+^ Calcd for C_10_H_15_NNaS_3_ 300.0157; Found 300.0156.

#### Preparation of **mCTA1**

To a dry ampule under N_2_ atmosphere, DEAm (6.00 g, 47 mmol), cyanomethyl dodecyl trithiocarbonate (200 mg, 630 μmol), AIBN (10.3 mg, 63 μmol) and 1,4-dioxane (12 mL) were added. The mixture was degassed by 3 freeze–pump–thaw cycles and the ampule was refilled with N_2_ atmosphere and sealed. The mixture was stirred in an oil bath thermostated at 65 °C for 3.5 h. After this time the reaction mixture was opened to air and cooled on dry ice. The solution was precipitated into petroleum ether (40–60 °C) to yield a yellow solid. The degree of polymerization was determined by ^1^H NMR spectroscopy by assessing the conversion of the monomer vinyl peak at 6.56 ppm to the peaks from the terminal alkyl peaks of the monomer and polymer at 1.50–0.92 ppm (Table 1). ^1^H NMR (400 MHz, CDCl_3_) *δ*/ppm: 3.71–2.89 (4H, br m, N(C**H**_2_CH_3_)_2_), 2.89–2.12 (1H, br m, C**H**CH_2_ of backbone), 2.12–1.45 (2H, br m, CHC**H**_2_ of backbone), 1.45–0.91 (6H, br m, N(CH_2_C**H**_3_)_2_). FT-IR (neat) *ν*/cm^–1^: 2975, 2934, 2879 (C–H stretch), 1631 (C=O), 1454, 1434 (C–H bend).

**Table 1:** Properties of the polymers studied in this work and in our previous report^60^

Polymer	Corona	*n*BA : DMA feed ratio	DP *n*BA^*b*^	DP DMA^*b*^	mol% *n*BA in core^*b*^	*M*_n NMR_ ^*b*^ (kg mol^–1^)	*M*_n SEC_ ^*c*^ (kg mol^–1^)	*Đ* ^*e*^	*R*_H_ ^*d*^ (nm)	*R*_core_ ^*e*^ (nm)
**mCTA1**	pDEAm	—	—	—	—	8.6^*a*^	7.7	1.11	—	—
**1**	pDEAm	1 : 1	19	19	50	12.9	12.0	1.16	7.3	3.4
**2**	pDEAm	7 : 3	22	11	67	12.5	12.0	1.14	10.6	4.5
**3**	pDEAm	4 : 1	25	8	76	12.6	13.0	1.16	11.9	5.0
**4**	pDEAm	9 : 1	24	4	86	12.1	12.4	1.14	11.4	5.1
**5**	pDEAm	1 : 0	34	0	100	13.0	13.7	1.16	14.7	6.3

**mCTA2**	pNIPAM	—	—	—	—	8.8	10.0	1.07	—	—
**6**	pNIPAM	1 : 1	20	17	54	12.9	13.0	1.10	8.3	3.5
**7**	pNIPAM	7 : 3	26	11	70	12.8	12.8	1.11	12.1	5.6
**8**	pNIPAM	4 : 1	27	7	79	12.4	12.7	1.10	13.4	6.2
**9**	pNIPAM	9 : 1	37	4	90	13.0	11.2	1.11	13.2	7.3
**10**	pNIPAM	1 : 0	40	0	100	13.9	10.9	1.11	17.5	8.1

**mCTA3**	pDEGMA	—	—	—	—	12.4	12.3	1.36	—	—
**11**	pDEGMA	1 : 1	16	19	46	16.4	15.8	1.41	8.4	2.7
**12**	pDEGMA	7 : 3	26	12	68	17.0	15.7	1.46	13.1	5.5
**13**	pDEGMA	4 : 1	27	8	77	17.0	18.3	1.37	13.7	5.6
**14**	pDEGMA	9 : 1	30	4	88	16.7	16.6	1.45	12.0	5.8
**15**	pDEGMA	1 : 0	40	0	100	17.6	14.3	1.41	14.6	6.9

**mCTA4**	pOEGMA	—	—	—	—	19.8	15.4	1.23	—	—
**16**	pOEGMA	1 : 1	17	22	44	24.2	20.1	1.34	9.0	3.1
**17**	pOEGMA	1 : 0	40	0	100	24.9	22.0	1.31	12.5	4.9

^*a*^ *M*_n_ calculated from conversion ^1^H NMR spectroscopy.

^*b*^Calculated using ^1^H NMR spectroscopy relative to the polymer end group (**mCTA2–4**) or known mCTA DP (**1–17**) (see Experimental section for details).

^*c*^Calculated from SEC analysis using 2% TEA in THF (**mCTA1**, **mCTA3–4**, **1–5** and **11–17**) or 5 mM NH_4_BF_4_ in DMF (**mCTA2** and **6–10**) as the eluent against poly(methyl methacrylate) standards.

^*d*^Calculated from multiple angle DLS analysis using the Stokes Einstein equation.

^*e*^Calculated from multiple angle SLS analysis (see ESI).

#### Preparation of **mCTA2**

To a dry ampule under N_2_ atmosphere, NIPAM (5.76 g, 51 mmol), cyanomethyl dodecyl trithiocarbonate (216 mg, 680 μmol), AIBN (11.2 mg, 68 μmol) and 1,4-dioxane (8.64 mL) were added. The mixture was degassed by 3 freeze–pump–thaw cycles and the ampule was refilled with N_2_ atmosphere and sealed. The mixture was stirred in an oil bath thermostated at 65 °C for 2 h. After this time the reaction mixture was opened to air and cooled on dry ice. The solution was precipitated into petroleum ether (40–60 °C) to yield a yellow solid. The degree of polymerization was determined by ^1^H NMR spectroscopy using integration of the signals at 3.33 and 4.00 ppm, attributed to the SC**H**_**2**_ of the chain transfer agent and the C**H**(CH_3_)_2_ of pNIPAM respectively (Table 1). ^1^H NMR (400 MHz, CDCl_3_) *δ*/ppm: 7.14–5.84 (br s, N**H**), 4.00 (1H, s, C**H**(CH_3_)_2_), 3.33 (br s, SC**H**_2_(CH_2_)_10_CH_3_ of end group), 2.49–1.96 (1H, br m, C**H**CH_2_ of backbone), 1.96–1.48 (2H, br m, CHC**H**_2_ of backbone), 1.38 (6H, br m, CH(C**H**_3_)_2_). FT-IR (neat) *ν*/cm^–1^: 3296 (N–H stretch), 2974, 2933 (C–H stretch), 1638 (C=O stretch), 1534 (N–H bend).

#### Preparation of **mCTA3**

To a dry ampule under N_2_ atmosphere, DEGMA (6.39 g, 34 mmol), 2-cyano-2-propyl dodecyl trithiocarbonate (155 mg, 450 μmol), AIBN (7.4 mg, 45 μmol) and 1,4-dioxane (9.6 mL) were added. The mixture was degassed by 3 freeze–pump–thaw cycles and the ampule was refilled with N_2_ atmosphere and sealed. The mixture was stirred in an oil bath thermostated at 65 °C for 18 h. After this time the reaction mixture was opened to air and cooled on dry ice. The solution was diluted with THF and purified by exhaustive dialysis against deionized water before lyophilization to yield a yellow, viscous oil. The degree of polymerization was determined by ^1^H NMR spectroscopy using integration of the signals at 3.20 and 4.09 ppm attributed to the SC**H**_**2**_ of the chain transfer agent and the C(O)OC**H**_2_ of pDEGMA respectively (Table 1). ^1^H NMR (400 MHz, CDCl_3_) *δ*/ppm: 4.09 (2H, br s, COOC**H**_2_CH_2_), 3.77–3.50 (6H, br m, OCH_2_C**H**_2_OC**H**_2_C**H**_2_), 3.38 (3H, br s, OC**H**_3_) 3.20 (br s, SC**H**_2_(CH_2_)_10_CH_3_ of end group), 2.11–1.66 (2H, br m, C(CH_3_)C**H**_2_ of backbone), 1.78–0.72 (3H, br m, C(C**H**_3_)CH_2_ of backbone). FT-IR (neat) *ν*/cm^–1^: 2934, 2879, 2828 (C–H stretch), 1727 (C=O), 1459 (C–H bend), 1111 (C–O stretch).

#### Preparation of **mCTA4**

To a dry ampule under N_2_ atmosphere, OEGMA (*M*_n_ = 300 g mol^–1^, 7.00 g, 23 mmol), methyl 4-cyano-4-(((ethylthio)carbonothioyl)thio) pentanoate (86 mg, 310 μmol), AIBN (5.1 mg, 31 μmol) and 1,4-dioxane (9.6 mL) were added. The mixture was degassed by sparging with argon for 15 min and sealed. The mixture was stirred in an oil bath thermostated at 65 °C for 8 h. After this time the reaction mixture was opened to air and cooled on dry ice. The solution was diluted with THF and purified by exhaustive dialysis against deionized water before lyophilization to yield a yellow, viscous oil. The degree of polymerization was determined by ^1^H NMR spectroscopy using integration of the signals at 2.47 and 4.07 ppm, attributed to the C**H**_**2**_C**H**_**2**_C(CN) of the chain transfer agent and the C(O)OC**H**_2_C**H**_**2**_ of pOEGMA respectively (Table 1). ^1^H NMR (400 MHz, CDCl_3_) *δ*/ppm: 4.07 (2H, br s, COOC**H**_2_CH_2_), 3.81–3.45 (br m, (OC**H**_2_C**H**_2_)_7–8_ of side chain), 3.36 (3H, br s, OC**H**_3_) 2.47 (br m, C**H**_2_C**H**_2_COOMe of end group), 2.13–1.59 (2H, br m, C(CH_3_)C**H**_2_ of backbone), 1.13–0.63 (3H, br m, C(C**H**_3_)CH_2_ of backbone). IR (neat) *ν*/cm^–1^: 2939, 2874, 2823 (C–H stretch), 1727 (C=O stretch), 1454 (C–H bend), 1100 (C–O stretch).

#### General procedure for the preparation of diblock copolymers **1–17**

Using the preparation of polymer **6** as an example, a general procedure for the preparation of diblock copolymers **1–15** is outlined as follows. Polymers **16–17** were prepared similarly but were degassed by sparging the mixture with argon prior to the heating. To a dry ampule under N_2_ atmosphere, **mCTA2** (800 mg, 91 μmol), *n*BA (234 mg, 1.83 mmol), DMA (180 mg, 1.82 mmol), AIBN (1.5 mg, 9.1 μmol) and 1,4-dioxane (2.5 mL) were added. The mixture was degassed by 3 freeze–pump–thaw cycles and the ampule was refilled with N_2_ atmosphere and sealed. The mixture was stirred in an oil bath thermostated at 65 °C for 190 min. After this time the reaction mixture was opened to air and cooled on dry ice. The polymer was precipitated into petroleum ether (40–60 °C) to yield a yellow solid. The degree of polymerization and ratio of *n*BA to DMA units were determined by ^1^H NMR spectroscopy, see below for details (Table 1). ^1^H NMR (300 MHz, CDCl_3_) *δ*/ppm: 7.14–5.84 (br s, N**H**), 4.00 (s, C**H**(CH_3_)_2_ of pNIPAM and C**H**_2_(CH_2_)_2_CH_3_ of p*n*BA), 3.33 (br s, SC**H**_2_(CH_2_)_10_CH_3_ end group), 3.22–2.77 (br m, CON(C**H**_3_)_2_ of pDMA) 2.77–2.00 (br m, C**H**CH_2_ of backbone), 1.98–1.49 (br m, CHC**H**_2_ of backbone and CH_2_C**H**_2_CH_2_CH_3_ of p*n*BA), 1.48–1.25 (CH_2_CH_2_C**H**_2_CH_3_ of p*n*BA), 1.38 (br m, CH(C**H**_3_)_2_ of pNIPAM), 0.93 (CH_2_CH_2_CH_2_C**H**_3_ of p*n*BA). IR (neat) *ν*/cm^–1^: 3296 (N–H stretch), 2974, 2933 (C–H stretch), 1736 (C=O stretch, ester), 1638 (C=O stretch, amide), 1534 (N–H bend). Owing to the overlap of the polymer peaks of polymers **1–5** with the end groups and the polymer peaks of **mCTA1**, the ratio of *n*BA to DMA for polymers **1–5** was performed using the normalized integrals of the corresponding peaks at 4.00 (p*n*BA) and 3.22–2.77 ppm (pDMA) after subtraction of the spectrum of **mCTA1** (see ESI†). Calculation of the ratio of *n*BA to DMA for polymers **6–10** was determined by comparison of the integral of the peak at 3.33 ppm (end group), with the integrals at 4.00 (pNIPAM + p*n*BA) and 3.22–2.77 ppm (pDMA). Calculation of the ratio of *n*BA to DMA for polymers **11–17** was performed using the integrals of the corresponding peaks at 4.00 (p*n*BA) and 3.22–2.77 ppm (pDMA) respectively, with respect to the known integral of the terminal methoxy protons at around 3.36 ppm at the end of the pDEGMA or pOEGMA side chain.

#### General procedure for the preparation of micelles by a solvent switch technique

In a typical experiment, the diblock copolymer (10 mg) was dissolved in acetone (2 mL) and allowed to stir for at least 40 min on ice. Ice cold deionized water (5 mL) was added to the stirred solution on ice using a peristaltic pump at a rate of 0.6 mL h^–1^. The homogeneous mixture was dialyzed exhaustively against deionized water (MWCO = 3–5 kDa). For polymers **1–10**, the dialysis was carried out at room temperature. For polymers **11–15**, it was necessary to remove the organic solvent by evaporation on ice under a flow of compressed air owing to the polymers lower LCST. For polymers **16** and **17**, both the addition of water and the removal of organic solvent by evaporation under a flow of compressed air were performed at room temperature owing to the polymers higher LCST. In each case, the final polymer concentration was made up to 1 mg mL^–1^ by dilution.

### Particle analysis

#### Turbidimetry

Analysis was performed at 1 mg mL^–1^ on a Perkin-Elmer Lambda 35 UV-vis instrument fitted with a Peltier heating and cooling system at a heating and cooling rate of 1 °C min^–1^. The transmittance at a wavelength of 500 nm was measured.

#### Light scattering

Data was collected using an ALV/CGS-3 Compact Goniometer System. d*n*/d*c* values were determined using a Shodex RI-101 refractometer. 1 mg mL^–1^ solutions were filtered through 0.45 μm nylon filters under a laminar flow hood prior to analysis at multiple angles from 50–150° against a toluene standard. The wavelength of the incident beam was 633 nm and for each angle, runs of at least 60 s were carried out.

For each micellar system, the resulting *g*_2_(*q*, *t*) autocorrelation functions from DLS analysis for each angle were analyzed by the REPES algorithm to determine a relaxation time, *τ*. The *τ* values at each angle were plotted against the square of the scattering wave vector, *q* to determine the apparent diffusion coefficient *D* according to eqn (1).^64,65^ Apparent hydrodynamic radii (*R*_H_) were then calculated using the Stokes Einstein eqn (2), where *η* is the solvent viscosity, *k*_B_ is the Boltzmann constant and *T* is the absolute temperature.
1

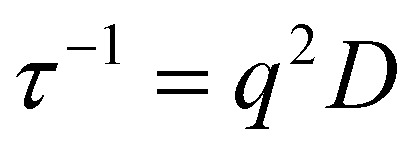



2

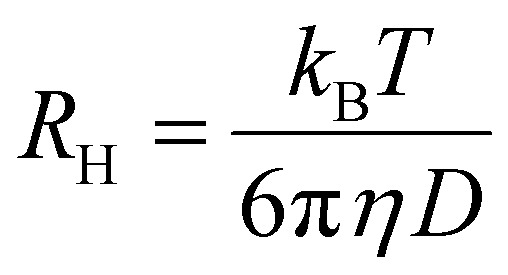




Using SLS analysis at the same angles, partial Zimm plots were obtained and the apparent aggregation number, *N*_agg_ for each set of micelles was calculated using eqn (3) and (4).^64,65^

3

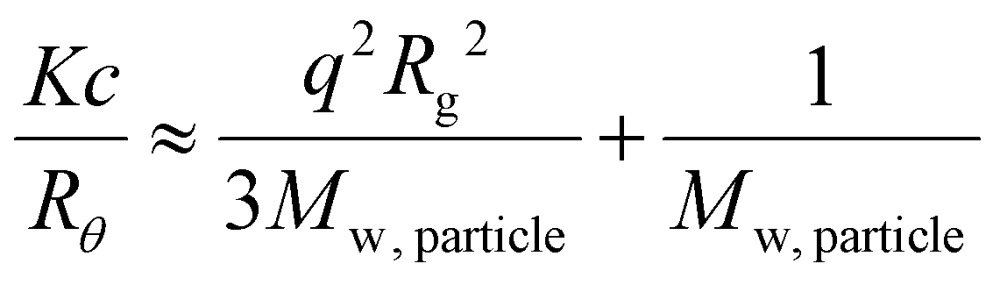



4

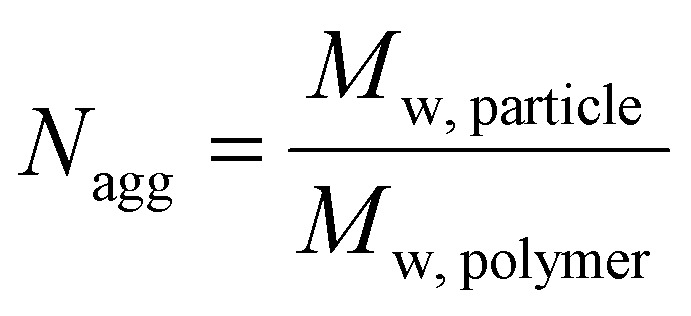




For SLS analysis, the intensity of the scattered light (*I*_sample_) was used to calculate *Kc*/*R*_*θ*_ for each angle, where *c* is the polymer concentration and *K* and *R*_*θ*_ are defined in the ESI.† It should be noted that since the radius of gyration (*R*_g_) of these micelles was less than 20 nm, the average value of *Kc*/*R*_*θ*_ over the angles analyzed was equal to the inverse of the particles’ molecular weight (*M*_w, particle_) and was used to calculate *N*_agg_. In the event of two relaxation modes being present, the REPES algorithm was used to determine the relative amplitudes of the fast and slow modes (*A*_fast_ and *A*_slow_). The scattering intensity contribution from each mode was determined using eqn (5) and (6).^65–67^

5

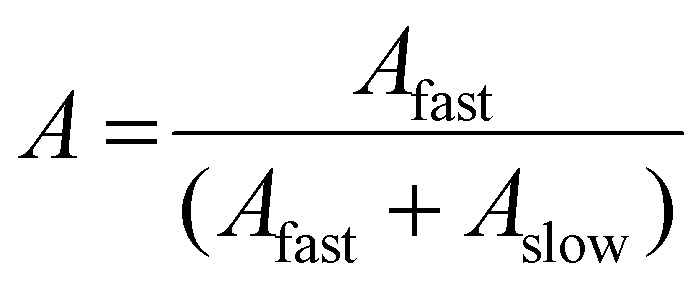



6

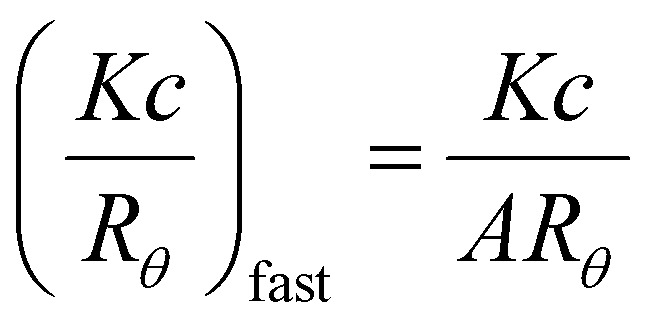




In the event of a data point from one observation angle falling outside of 10% error of *Kc*/*R*_*θ*_, the point was excluded from the average in the calculation of *M*_w, particle_. For additional calculations relating to the light scattering data, see ESI.† Polymers **1–10** and **16–17** were analyzed at 20 °C, however because of the lower transition temperature of the pDEGMA-stabilized micelles, polymers **11–15** were analyzed at 10 °C.

## Results and discussion

### Diblock copolymer synthesis and micelle preparation

A series of diblock copolymers with varying degrees of core hydrophobicity were synthesized by RAFT polymerization for each of the coronas investigated. In each case, the corona-forming block was synthesized first (**mCTA1–4**) such that each of the diblock copolymers in the series had identical corona lengths and molecular weight distributions (Table 1 and Scheme 2). For **mCTA2** and **mCTA3**, the methylene protons adjacent to the trithiocarbonate group of the ω-end group could be observed in the ^1^H NMR spectrum, and so it was possible to calculate *M*_n_ by NMR spectroscopy (*M*_n, NMR_) by comparing the integrals of the corresponding proton peaks to the integrals of the proton peaks on the polymer side chain. For **mCTA4**, *M*_n, NMR_ was calculated using the integrals of the four protons from the two methylene groups on the α-end group relative to the integrals of the side chain proton peaks. For **mCTA1**, it was not possible to recognize any peaks from the end group protons in the ^1^H NMR spectrum so the *M*_n, NMR_ was calculated from the conversion of monomer to polymer taken after the polymerization. This number (8600 g mol^–1^) is in good agreement with triple detection SEC, which calculated an *M*_n_ of 8100 g mol^–1^. Chain extension of the macro chain transfer agents yielded the amphiphilic diblock copolymers (**1–17**, Scheme 2). The degree of hydrophobicity of each of the copolymers was able to be varied by altering the monomer feed ratios of permanently hydrophilic DMA and hydrophobic *n*BA. It can be seen in Table 1 and Fig. S4, S7, S10 and S13† that for each of the diblock copolymers there is an increase in the *M*_n, SEC_ relative to the respective mCTAs, whilst the dispersity does not increase significantly after the extension. The percentage *n*BA of each of the core-forming blocks, determined by ^1^H NMR spectroscopy, was also found to be similar to the initial monomer feeds. As has been discussed previously, the *n*BA and DMA units in the core are randomly incorporated into the core-forming block as predicted by the reactivity ratios of the two monomers.^68^ Because of this phenomenon, we expect there to be no phase separation between the incompatible units in the core, and that the core-forming block can be thought of as having a uniform hydrophobicity determined by its composition. Once the four series of diblock copolymers were achieved, each of them were self-assembled into micellar structures using a solvent switch technique from acetone into ice cold water (see Experimental section for more details).

### Multi-angle light scattering analysis

**Fig. 2:**
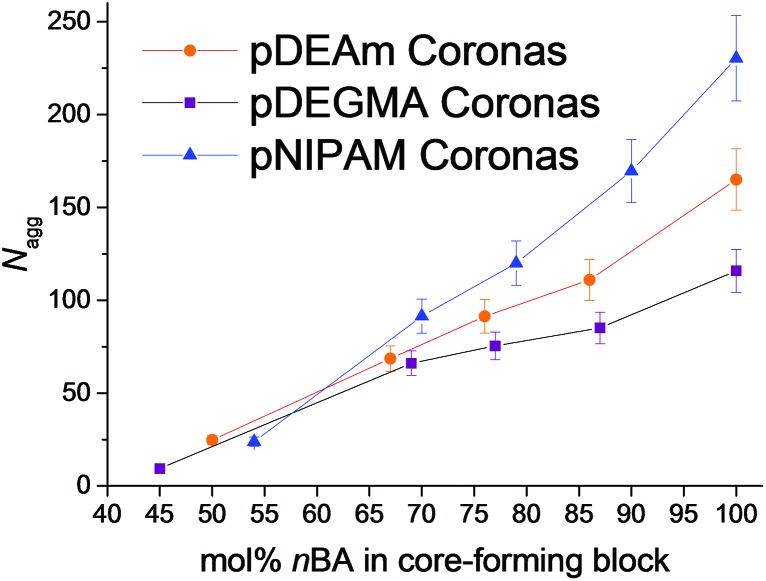
Variation in the particles’ *N*_agg_ with the molar fraction of hydrophobic *n*BA in the core-forming block, as determined by SLS analysis. Micelles with pDEAm (polymers **1–5**, orange circles), pNIPAM (polymers **6–10**, blue triangles) and pDEGMA coronas (polymers **11–15**, purple squares) are shown. Error bars represent 10% error.

The resultant particles were analyzed using a combination of static and dynamic light scattering at multiple angles (SLS and DLS, respectively). DLS gave information on the particles sizes, specifically their hydrodynamic radii (*R*_H_) in solution, whereas SLS gave the average molecular weights of the particles (*M*_w, particle_) and the radii of the particles’ cores (*R*_core_). The two radii for each micelle are outlined in Table 1. As can be seen in Fig. S15–S18,† for each particle, their relaxation time determined by DLS showed a *q* ^2^ dependence indicative of Brownian motion and their partial Zimm plots returned a negligible slope owing to the micelles’ low expected *R*_g_ values. Additional calculations were performed on the SLS data to give *N*_agg_ (see Experimental section and ESI†). For each of the micellar series, the *N*_agg_ and therefore *R*_core_ increased as a function of the percentage composition of *n*BA in the core-forming block (Fig. 2 and Table 1). This can be understood by the necessity of highly hydrophobic moieties to shield themselves from the aqueous environment, as described by the hydrophobic effect. The increase in both *N*_agg_ and *R*_core_ with increasing hydrophobicity results in there being a lower overall number of particles in solution. This is energetically favorable as it leads to a reduction in the total interfacial area between the hydrophobic core and the solvent, and therefore a lower interfacial energy.^45^ The results from each of the series of micelles demonstrate that increasing the percentage composition of hydrophobic monomers in the core-forming block is a robust method for synthesizing micelles with tunable *N*_agg_, along with other reports in the literature where variation in the polymer's ionization,^67^ core to corona block length ratio,^45^ and addition of hydrophobic homopolymer^69^ yield similar results.

### Thermoresponsive behavior

#### Effect of core hydrophobicity on reversibility

**Fig. 3:**
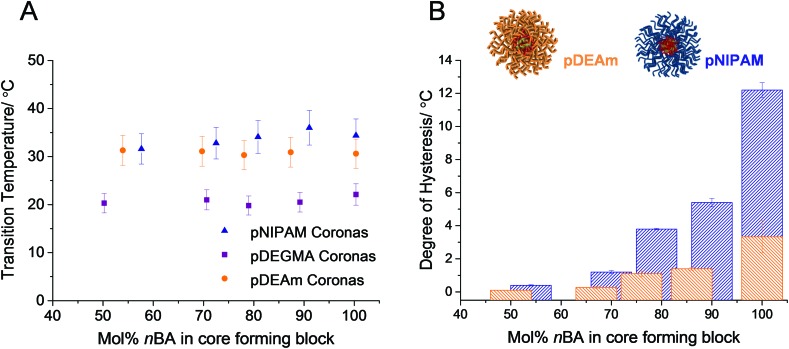
A: Variation of the cloud point transition temperatures of polymers **1–5** (orange circles), **6–10** (blue triangles) and **11–15** (purple squares) with the molar fraction of hydrophobic *n*BA in the core-forming block, as determined by turbidimetry. Error bars represent 10% error. B: Variation of thermal hysteresis of micelles comprised of pDEAm coronas, which cannot form hydrogen bonds between polymer chains (polymers **1–5**, orange bars), and pNIPAM coronas, which can (polymers **6–10**, blue bars). Values determined by turbidimetry and plotted as a function of mol% *n*BA in the core-forming block. Error bars represent the standard deviation across 3 repeats.

Upon heating each of the micellar systems, macroscopic precipitation occurred as a result of the corona forming polymer–polymer interactions in preference to polymer–solvent interactions. The cloud point, defined here as the temperature at which the amount of transmitted light drops to half of the normalized maximum transmitted light, was measured by variable temperature turbidimetry. Note that this temperature is distinct from the LCST as only one concentration was measured. The LCST is defined as the minimum point on the binodal curve of the phase diagram, whereas the observable cloud point is a macroscopic effect that occurs as a result of the phase separation associated with LCST phase behavior. As such, the two terms should not be used interchangeably.^19^ As was previously reported for polymers **6–10**,^60^ the cloud points of polymers **1–17** were not dependent on the mol% *n*BA in the core-forming block (Fig. 3A and Table 2), and therefore did not show a correlation with *N*_agg_.

**Table 2:** *N*_agg_ and turbidimetry data for polymers **11–17**

Polymer	Corona	Mol% *n*BA in core block	*N*_agg_	Cloud point/°C	Degree of hysteresis/°C
**11**	**mCTA3**	46	9	20.3^*a*^ ^,^ ^*d*^	0.4
**12**	**mCTA3**	68	66	21.0^*a*^ ^,^ ^*d*^	0.5
**13**	**mCTA3**	77	75	19.8^*a*^ ^,^ ^*d*^	0.1
**14**	**mCTA3**	88	85	20.5^*b*^ ^,^ ^*d*^	Irreversible^*c*^
**15**	**mCTA3**	100	116	22.1^*b*^ ^,^ ^*d*^	Irreversible^*c*^

**16**	**mCTA4**	44	12	61.1^*a*^ ^,^ ^*e*^	1.1
**17**	**mCTA4**	100	33	62.5^*b*^ ^,^ ^*e*^	Irreversible^*c*^

^*a*^Mean cloud point upon heating the micellar solutions determined using turbidimetry data across from three heating and cooling cycles.

^*b*^For micelles with irreversible transitions, the cloud point from the first heating cycle is shown.

^*c*^The degree of hysteresis from solutions exhibiting irreversible phase transitions was not determined as the normalized transmittance did not reach 0.5 in the cooling cycle.

^*d*^Heated from 10–40 °C.

^*e*^Heated from 50–95 °C.

It was noted that some of the micelles in each series exhibited a thermal hysteresis, whereby the reversibility of the transition was slow. The degree of hysteresis, defined here as the difference in the cloud points upon heating and upon cooling the micelles, increased as a function of mol% *n*BA in the core-forming block, for each of the micellar systems **1–10** (Fig. 3B). This was attributed to differences in the hydration of the micellar cores in solution. For instance micelles of polymer **1** have a higher degree of core hydration than micelles of polymer **5**, owing to the fact that there is a larger mol% of hydrophilic DMA in its core-forming block. By this virtue, upon heating the micelles, those comprised of polymer **1** form precipitates that are more hydrated than those of polymer **5**. In turn, this facilitates the rapid redissolution of micelles with moderately hydrophobic cores, compared with those with a highly hydrophobic character.^60^ It is therefore important to note that although pDEAm has been successfully designed in the literature to yield homopolymers with an LCST close to pNIPAM but with negligible hysteresis, when incorporated into a micellar structure, the composition of the core-forming block is as important as the design of the corona-forming block when considering hysteresis.

#### Effect of hydrogen bond donor ability on reversibility

By comparing micelles across the different linear corona systems, those with pNIPAM (**6–10**) and pDEAm (**1–5**) coronas, the effect of hydrogen bond donating ability of the polymer side-chain can be elucidated (Fig. 1). pNIPAM has a monosubstituted amide in its side-chain and therefore it can participate in hydrogen bonding interactions as both a donor and an acceptor, whereas pDEAm has a disubstituted amide so it can only participate in hydrogen bonding as an acceptor. This means that in the single-phase temperature regime, both polymers can participate in hydrogen bonding with the aqueous solvent. However, above the LCST, in the biphasic temperature regime, pNIPAM can form hydrogen bonding interactions between polymer chains in its globular structure, and between side groups in an intramolecular fashion, whereas pDEAm is unable to form polymer–polymer hydrogen bonding interactions. The two polymers can be considered to be analogous in most other aspects as they both exhibit a high *T*_g_ and have almost identical cloud points. Considering the turbidimetry data for both series of micelles in Fig. 3B, S19 and S20,† it is evident that the hydrogen bonding ability of the corona is an important factor in determining the rehydration rate of the micelles. Micelles of polymers **1–5** exhibit much faster rehydration rates, and therefore a lower degree of hysteresis, than micelles of polymers **6–10** of comparable mol% *n*BA. This has been studied for pDEAm^61^ and other thermoresponsive unimeric homopolymer chains with no hydrogen bond donating ability,^27–33^ and is understood by the rationale that polymers **1–5** form weaker polymer–polymer interactions above the transition temperature. As discussed previously, the strong polymer–polymer hydrogen bonding interactions present in the globular state of pNIPAM chains leads to a non-perfect globule-to-coil transition in the cooling cycle, at temperatures close to the transition temperature.^35^ This leads to slower rehydration kinetics and a greater degree of hysteresis.

It can be seen in Fig. 3B that this effect is exaggerated when the chains are no longer homopolymers and are instead covalently grafted from a hydrophobic micellar core. For example, the difference in hysteresis between the loosely packed micelles in each series (polymers **1** and **6**) is much smaller than the difference in hysteresis between densely packed micelles with more hydrophobic cores (polymers **5** and **10**). This is a result of differences in core hydration, as discussed previously. This finding demonstrates that design of polymers with a lower degree of thermal hysteresis on the whole is possible by limiting the strength of the polymer–polymer interactions through changing the hydrogen bonding ability. However, caution should be taken when designing these polymers for a specific application as the introduction of some degree of thermal hysteresis will occur in self-assemblies with very hydrophobic cores, owing to the hydration effects discussed in the previous section.

#### Effect of corona chain architecture on reversibility

**Fig. 4:**
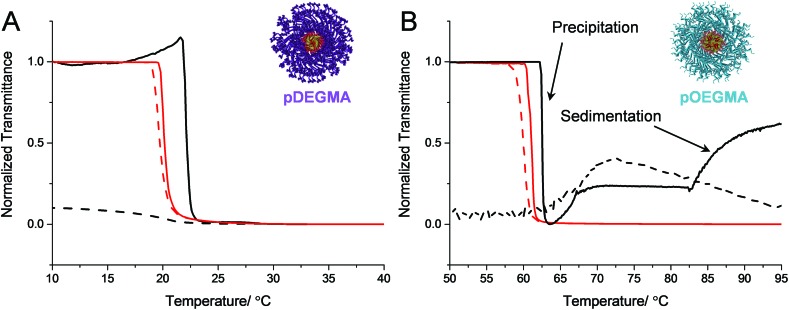
Turbidimetry analyses of micelles with pDEGMA (A) and pOEGMA coronas (B). In each case, solid lines represent heating cycles and dashed lines represent cooling cyles. A shows turbidimetry curves for polymers **11** (red) and **15** (black). B shows turbidimetry curves for polymers **16** (red) and **17** (black). For clarity, some instances of macroscopic precipitation and sedimentation have been labelled and the cooling curve for polymer **17** has been smoothed.

Poly(oligo(ethylene glycol) methacrylate)s have also gathered interest in recent years as an attractive alternative to pNIPAM. They show LCST-type phase behavior and have cloud points that can be tuned by either varying the ethylene glycol side chain length or by copolymerization of two monomers containing different oligo(ethylene glycol) side chain lengths.^27,29^ Contrary to linear pNIPAM and pDEAm, these have brush-like chain architectures and so we postulated that altering the confinement of the polymer chains in the micellar structure could have an effect on their thermoresponsive behavior. Therefore, we used thermoresponsive micelles with tunable *N*_agg_ to assess the effect of corona chain architecture on the phase behavior. In this respect, pDEGMA (**mCTA3**) and pOEGMA (**mCTA4**) macroCTAs were synthesized and used as the hydrophilic corona blocks in the micellar structures of their corresponding diblock copolymers (**11–15** and **16–17** respectively). Contrary to **mCTA1** and **mCTA2**, these coronas exhibit low a *T*_g_ so were initially expected to show minimal hysteresis in their thermal transitions. Additionally, neither **mCTA3** nor **mCTA4** can participate in polymer–polymer hydrogen bonding interactions in their globular states (Fig. 1). It was found that micelles with brush-like coronas, namely those comprised of polymers **11–17**, exhibited different behavior to those whose corona is comprised of linear polymers (**1–10**). Considering micelles with pDEGMA coronas (**11–15**), it can be seen in Fig. 4A and Table 2 that at low mol% *n*BA, the micelles show a fully reversible transition with almost negligible thermal hysteresis (**11–13**), owing to the reasons discussed above. However, as the mol% *n*BA was increased above 88% *n*BA in the core-forming block (**14** and **15**), it was observed in the turbidimetry analysis that after the first heating cycle, the solution remained turbid throughout the rest of the heating and cooling cycles. In these cases, the macroscopic precipitation was completely irreversible even on the timescale of several months at 4 °C. This result led us to two possible conclusions, one being that because pDEGMA had a low cloud point transition temperature of around 20 °C, the polymer chains were not hydrophilic enough to undergo reversible phase transitions once the hydrophobic core-forming block was introduced. Therefore at a critical core hydrophobicity, the micelles were too destabilized and undergo irreversible transitions. This conclusion seemed unlikely since the cloud point of the micelles did not change dramatically across the series and so it was unlikely that the core-forming block had a major effect on the corona-forming block's ability to stabilize the micelles directly. The second possible conclusion was that the pDEGMA micelles exhibited markedly different behavior at high *N*_agg_ as a result of increased chain entanglements occurring at some critical core hydrophobicity. This difference in chain confinement is demonstrated in Fig. S25,† whereby the number of chains packed into the volume of the micelle increases as a function of core hydrophobicity. It can be rationalized that at a critical *N*_agg_, the coronal chain entanglements become such that the corona chains are no longer able to redisperse the micelles back into the solution on the timescale of the experiment. In order to test which of the two arguments was the most compelling, a further series of micelles, whose coronas consisted of pOEGMA were synthesized (**16** and **17**) and their thermoresponsive behavior assessed. These micelles had a corona that was more hydrophilic than the pDEGMA micelles (**11–15**) as evidenced by its higher cloud point temperature, outlined in Table 2. If the first conclusion was the most likely, the pOEGMA micelles would display fully reversible transitions similar to micelles of polymers **1–10**, as these would be hydrophilic enough to facilitate redissolution even after the introduction of a hydrophobic core-forming block. In contrast, the pOEGMA coronas have a longer side chain than the pDEGMA coronas (4–5 ethylene glycol units per monomer *vs*. 2 ethylene glycol units respectively) and as such the pOEGMA coronas can be considered as having more of a brush-like architecture. This further deviation from a linear architecture should mean that the polymer chains are more confined in the micellar structure and so these should also undergo irreversible phase transitions if the second conclusion was the likely mechanism behind the irreversible phase transitions. By assessing the thermoresponsive behavior of the pOEGMA micelles, it is clear that micelles with cores composed solely of *n*BA (**17**) undergo an irreversible phase transition (Fig. 4B and Table 2). Note that the fluctuation in transmittance in the cooling cycle is a result of not only precipitation but also sedimentation of the sample to the bottom of the cuvette with time. In contrast, micelles whose cores were only comprised of 44% *n*BA (**16**), showed fully reversible phase transitions in a similar manner to polymers **11–13**. These micelles had an *N*_agg_ of 12 so were very loosely packed in comparison to micelles comprised of polymer **17** (*N*_agg_ = 33) and showed a similar thermal hysteresis to the homopolymer, **mCTA4** (data not shown). These results suggests that the irreversible phase transitions observed in micelles with pOEGMA and pDEGMA coronas can likely be attributed to the increased entanglement of a polymer with a brush-like architecture at a critical chain confinement, rather than pDEGMA's relatively low hydrophilicity.

As the cloud point measurements for polymers **16–17** were performed at a much higher temperature than polymers **11–15**, it was postulated that hydrolysis of the *n*BA ester side-chain could have occurred, which may have led to irreversible phase separation. SEC analysis revealed that polymer **17**, recovered by lyophilization, had an essentially identical molecular weight distribution before and after three heating cycles indicating that the polymer was unchanged by the turbidimetry analysis (Fig. S14†). It should be noted that upon heating micelles comprised of polymer **17** to just 70 °C, rather than 95 °C in the initial study, a reversible phase transition with a degree of hysteresis of roughly 2 °C was observed (Fig. S23†). However, when cycling the heating and cooling experiments the micelles showed poor reversibility. This finding indicated that the polymer can entangle and potentially rearrange in the precipitated bulk when heated for long periods, which leads to irreversible transitions, however the rearrangement of the chains is hindered when heated for just short periods. The fact that pOEGMA and pDEGMA have *T*_g_ values lower than their transition temperature means the chains have the mobility to achieve this in the precipitated form, in contrast to the pDEAm and pNIPAM micelles, whose coronal chains are vitrified and glassy above the transition temperature.

To the best of our knowledge, the reversibility of the thermal transition of block copolymer micelles with pOEGMA or pDEGMA coronas has not been widely reported. However, the results are in concordance with Armes and Zheng *et al.* who showed that polypyrrole nanoparticles stabilized using a pOEGMA corona exhibited irreversible phase transitions upon either heating the solution, or irradiating the nanoparticles with near infra-red light to induce photothermal aggregation.^70^ Here, the irreversible transition was attributed to the high Hamaker constant of the polypyrrole cores, indicative of strong van der Waals’ interactions between the cores in the particles’ aggregated state. This may explain to some extent why this behavior is only observed in highly hydrophobic micellar cores. However, the fact that micelles comprised of polymers with linear coronas (**1–10**) exhibited reversible phase transitions for all of the core compositions investigated indicates that the brush-like structure of pOEGMA and pDEGMA, coupled with their low *T*_g_, plays a role in limiting the reversibility of micelles comprised of polymers **11–17**.

This important finding highlights that the micellar tethering of the corona chains leads to behavior that is drastically different from that of unimeric homopolymers. Although poly(ethylene glycol (meth)acrylate) based thermoresponsive polymers have been hailed as tunable smart materials with low thermal hysteresis, this reversible behavior may be limited to non- or loosely assembled micellar structures with low degrees of chain entanglement. Indeed, for certain applications, an irreversible phase transition is actually the desired outcome, for instance when designing polymers to permanently block blood vessels at the site of a tumor, or for sensors or logic gates with a memory of their thermal history. As such, the design and consideration of the behavior of an entire formulation is critical as they typically display behavior very different from their constituent parts.

## Conclusions

Well-defined, responsive, amphiphilic block copolymers containing four different thermoresponsive corona blocks were synthesized by RAFT polymerization and assembled into micellar structures in aqueous media. Multi-angle DLS and SLS revealed that the micelles from each series had tunable *N*_agg_ and this variation was used to probe the effects of altering the corona chemistry, chain confinement and core hydrophobicity on the thermoresponsive behavior, specifically the degree of hysteresis, of the micelles. Turbidimetry analysis revealed that higher core hydrophobicities led to a higher degree of hysteresis across each micellar series, owing to differences in core hydration. As expected, linear corona chains that could form polymer–polymer hydrogen bonding interactions (pNIPAM) showed a greater hysteresis than those that could not (pDEAm). Finally brush-like coronas (pDEGMA and pOEGMA) showed irreversible phase transitions at high core hydrophobicity and *N*_agg_. It was postulated that this was owing to increased coronal chain entanglements or rearrangement, which prevented micellar redissolution. These results highlight the complexity of hysteresis in thermoresponsive polymer systems, and that responsive self-assembled polymer structures do not necessarily exhibit behavior analogous to the sum of their respective unimer chains. Therefore, it is important to consider the effects of the chemistry and the architecture of individual polymer chains, as well as the structure of the resultant assembled particle when predicting the ultimate phase behavior of a given formulation, and when designing these formulations for a desired application.

## References

[cit1] Akimoto J., Nakayama M., Okano T. (2014). J. Controlled Release.

[cit2] Bastakoti B. P., Guragain S., Nakashima K., Yamauchi Y. (2015). Macromol. Chem. Phys..

[cit3] Bawa P., Pillay V., Choonara Y. E., du Toit L. C. (2009). Biomed. Mater..

[cit4] Lai W.-F., Shum H. C. (2016). Nanoscale.

[cit5] Louage B., Zhang Q., Vanparijs N., Voorhaar L., Vande Casteele S., Shi Y., Hennink W. E., Van Bocxlaer J., Hoogenboom R., De Geest B. G. (2015). Biomacromolecules.

[cit6] Mohapatra H., Kim H., Phillips S. T. (2015). J. Am. Chem. Soc..

[cit7] Kelley E. G., Albert J. N. L., Sullivan M. O., Epps III T. H. (2013). Chem. Soc. Rev..

[cit8] Phillips D. J., Prokes I., Davies G.-L., Gibson M. I. (2014). ACS Macro Lett..

[cit9] Reineke T. M. (2016). ACS Macro Lett..

[cit10] Peng Y., Jiang X., Chen S., Wu Q., Shen J., Wu W. (2015). Polym. Chem..

[cit11] Phillips D. J., Davies G.-L., Gibson M. I. (2015). J. Mater. Chem. B.

[cit12] Cao P.-F., Mangadlao J. D., Advincula R. C. (2015). Polym. Rev..

[cit13] Jochum F. D., Theato P. (2013). Chem. Soc. Rev..

[cit14] Schmaljohann D. (2006). Adv. Drug Delivery Rev..

[cit15] Cabane E., Zhang X., Langowska K., Palivan C., Meier W. (2012). Biointerphases.

[cit16] Stuart M. A. C., Huck W. T. S., Genzer J., Muller M., Ober C., Stamm M., Sukhorukov G. B., Szleifer I., Tsukruk V. V., Urban M., Winnik F., Zauscher S., Luzinov I., Minko S. (2010). Nat. Mater..

[cit17] Gil E. S., Hudson S. M. (2004). Prog. Polym. Sci..

[cit18] Theato P., Sumerlin B. S., O'Reilly R. K., Epps III T. H. (2013). Chem. Soc. Rev..

[cit19] Gibson M. I., O'Reilly R. K. (2013). Chem. Soc. Rev..

[cit20] Roy D., Brooks W. L. A., Sumerlin B. S. (2013). Chem. Soc. Rev..

[cit21] Seuring J., Agarwal S. (2012). Macromol. Rapid Commun..

[cit22] Akimoto J., Nakayama M., Sakai K., Okano T. (2009). Biomacromolecules.

[cit23] Ward M. A., Georgiou T. K. (2011). Polymer.

[cit24] Jeong B., Kim S. W., Bae Y. H. (2002). Adv. Drug Delivery Rev..

[cit25] Yu L., Ding J. (2008). Chem. Soc. Rev..

[cit26] Schild H. G. (1992). Prog. Polym. Sci..

[cit27] Lutz J.-F. (2008). J. Polym. Sci., Part A: Polym. Chem..

[cit28] Lutz J.-F., Akdemir Ö., Hoth A. (2006). J. Am. Chem. Soc..

[cit29] Lutz J.-F., Hoth A. (2006). Macromolecules.

[cit30] Hoogenboom R., Thijs H. M. L., Jochems M. J. H. C., van Lankvelt B. M., Fijten M. W. M., Schubert U. S. (2008). Chem. Commun..

[cit31] Okamura H., Mori T., Minagawa K., Masuda S., Tanaka M. (2002). Polymer.

[cit32] Samanta S., Bogdanowicz D. R., Lu H. H., Koberstein J. T. (2016). Macromolecules.

[cit33] Ieong N. S., Redhead M., Bosquillon C., Alexander C., Kelland M., O'Reilly R. K. (2011). Macromolecules.

[cit34] Cheng H., Shen L., Wu C. (2006). Macromolecules.

[cit35] Wu C., Wang X. (1998). Phys. Rev. Lett..

[cit36] Ding Y., Ye X., Zhang G. (2005). Macromolecules.

[cit37] Lu Y., Zhou K., Ding Y., Zhang G., Wu C. (2010). Phys. Chem. Chem. Phys..

[cit38] Aseyev V., Tenhu H. and Winnik F. M., in Self Organized Nanostructures of Amphiphilic Block Copolymers II, ed. H. E. A. Müller and O. Borisov, Springer Berlin Heidelberg, Berlin, Heidelberg, 2011, pp. 29–89.

[cit39] Van Durme K., Van Assche G., Van Mele B. (2004). Macromolecules.

[cit40] Seuring J., Agarwal S. (2012). Macromolecules.

[cit41] Matyjaszewski K., Xia J. (2001). Chem. Rev..

[cit42] Moad E. R. G., Thang S. (1998). Macromolecules.

[cit43] Keddie D. J. (2014). Chem. Soc. Rev..

[cit44] Cameron N. S., Corbierre M. K., Eisenberg A. (1999). Can. J. Chem..

[cit45] Mai Y., Eisenberg A. (2012). Chem. Soc. Rev..

[cit46] Blanazs A., Armes S. P., Ryan A. J. (2009). Macromol. Rapid Commun..

[cit47] Yan Q., Zhou R., Fu C., Zhang H., Yin Y., Yuan J. (2011). Angew. Chem., Int. Ed..

[cit48] Tamate R., Ueki T., Yoshida R. (2014). Adv. Mater..

[cit49] Meng F., Zhong Z., Feijen J. (2009). Biomacromolecules.

[cit50] Doncom K. E. B., Hansell C. F., Theato P., O'Reilly R. K. (2012). Polym. Chem..

[cit51] Feng A., Zhan C., Yan Q., Liu B., Yuan J. (2014). Chem. Commun..

[cit52] Cai Y., Aubrecht K. B., Grubbs R. B. (2011). J. Am. Chem. Soc..

[cit53] Sundararaman A., Stephan T., Grubbs R. B. (2008). J. Am. Chem. Soc..

[cit54] Zayas H. A., Lu A., Valade D., Amir F., Jia Z., O'Reilly R. K., Monteiro M. J. (2013). ACS Macro Lett..

[cit55] Moughton A. O., O'Reilly R. K. (2010). Chem. Commun..

[cit56] Moughton A. O., Patterson J. P., O'Reilly R. K. (2011). Chem. Commun..

[cit57] Wei K., Su L., Chen G., Jiang M. (2011). Polymer.

[cit58] Wang X., Li L. (2016). Polymer.

[cit59] Wang W., Gao C., Qu Y., Song Z., Zhang W. (2016). Macromolecules.

[cit60] Blackman L. D., Wright D. B., Robin M. P., Gibson M. I., O'Reilly R. K. (2015). ACS Macro Lett..

[cit61] Idziak I., Avoce D., Lessard D., Gravel D., Zhu X. X. (1999). Macromolecules.

[cit62] Abel B. A., McCormick C. L. (2016). Macromolecules.

[cit63] Convertine A. J., Benoit D. S. W., Duvall C. L., Hoffman A. S., Stayton P. S. (2009). J. Controlled Release.

[cit64] Schärtl W., Light Scattering from Polymer Solutions and Nanoparticle Dispersions, Springer, 2007.

[cit65] Patterson J. P., Robin M. P., Chassenieux C., Colombani O., O'Reilly R. K. (2014). Chem. Soc. Rev..

[cit66] Patterson J. P., Kelley E. G., Murphy R. P., Moughton A. O., Robin M. P., Lu A., Colombani O., Chassenieux C., Cheung D., Sullivan M. O., Epps T. H., O'Reilly R. K. (2013). Macromolecules.

[cit67] Wright D. B., Patterson J. P., Pitto-Barry A., Cotanda P., Chassenieux C., Colombani O., O'Reilly R. K. (2015). Polym. Chem..

[cit68] Neugebauer D., Matyjaszewski K. (2003). Macromolecules.

[cit69] Zhang L., Eisenberg A. (1996). J. Am. Chem. Soc..

[cit70] Au K. M., Chen M., Armes S. P., Zheng N. (2013). Chem. Commun..

