# Supplementary material for: Probing the causes of thermal hysteresis using tunable N agg micelles with linear and brush-like thermoresponsive coronas
Source: Polym Chem. 2016 Aug 9;8(1):233–44. doi: 10.1039/c6py01191h (PMC5361139; doi:10.1039/c6py01191h)
Supplement: Supplementary file 1 [file PY-008-C6PY01191H-s001.pdf]

Electronic Supplementary Material (ESI) for Polymer Chemistry.
This journal is © The Royal Society of Chemistry 2016

Supplementary Information

"Probing the Causes of Thermal Hysteresis Using Tunable N_{agg} Micelles with Linear and Brush-Like Thermoresponsive Coronas"

Lewis D. Blackman,[a] *Matthew I. Gibson*[a,b]* *and Rachel K. O'Reilly*[a]*

[a] *Dept. of Chemistry, University of Warwick, Gibbet Hill Road, Coventry, CV4 7AL, UK*

[b] *Warwick Medical School, University of Warwick, Gibbet Hill Road, Coventry, CV4 7AL, UK*

Contents

Supplementary characterization data 2

- NMR spectra of methyl 4-cyano-4-(((ethylthio)carbonothioyl)thio) pentanoate 2
- Representative polymer characterization data 3
- Representative light scattering data 12
- Representative turbidimetry data 16

Additional calculations and discussions 21

- Calculation of core composition for polymers 1-5 21
- Definitions and calculations regarding the light scattering data 22
- Discussion of the effect of chain density on reversibility for pDEGMA micelles 23

Supplementary characterization data

NMR spectra of methyl 4-cyano-4-(((ethylthio)carbonothioyl)thio) pentanoate

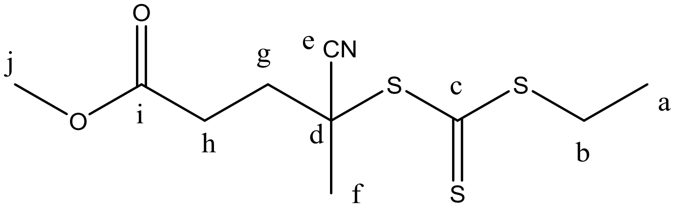

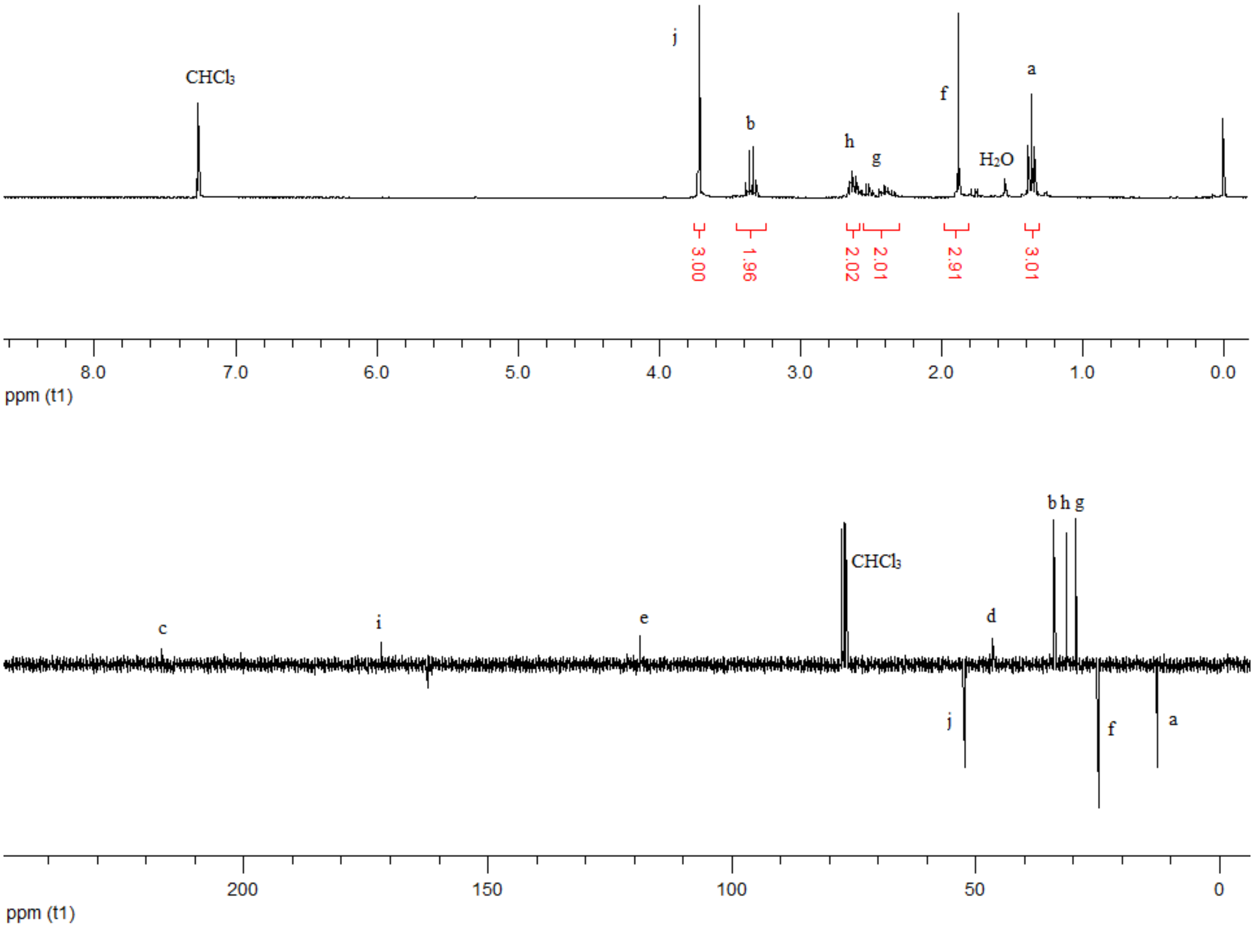

Figure S1. ^{1}H (above) and ^{13}C DEPT (below) NMR spectra of methyl 4-cyano-4-(((ethylthio)carbonothioyl)thio) pentanoate, analyzed at 300 and 75 MHz respectively, in $CDCl_3$.

Representative polymer characterization data

^{1}H NMR and SEC data for the mCTA and the 50 mol% *n*BA diblock copolymer in each series is shown below

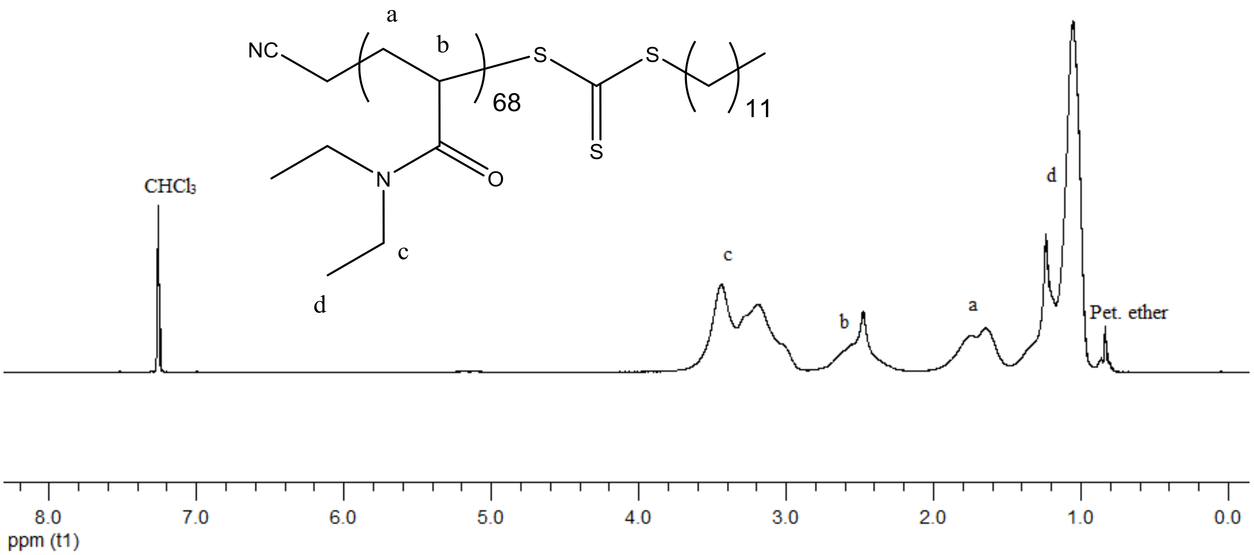

Figure S2. ^{1}H NMR spectrum of **mCTA1**, analyzed at 400 MHz in $CDCl_3$.

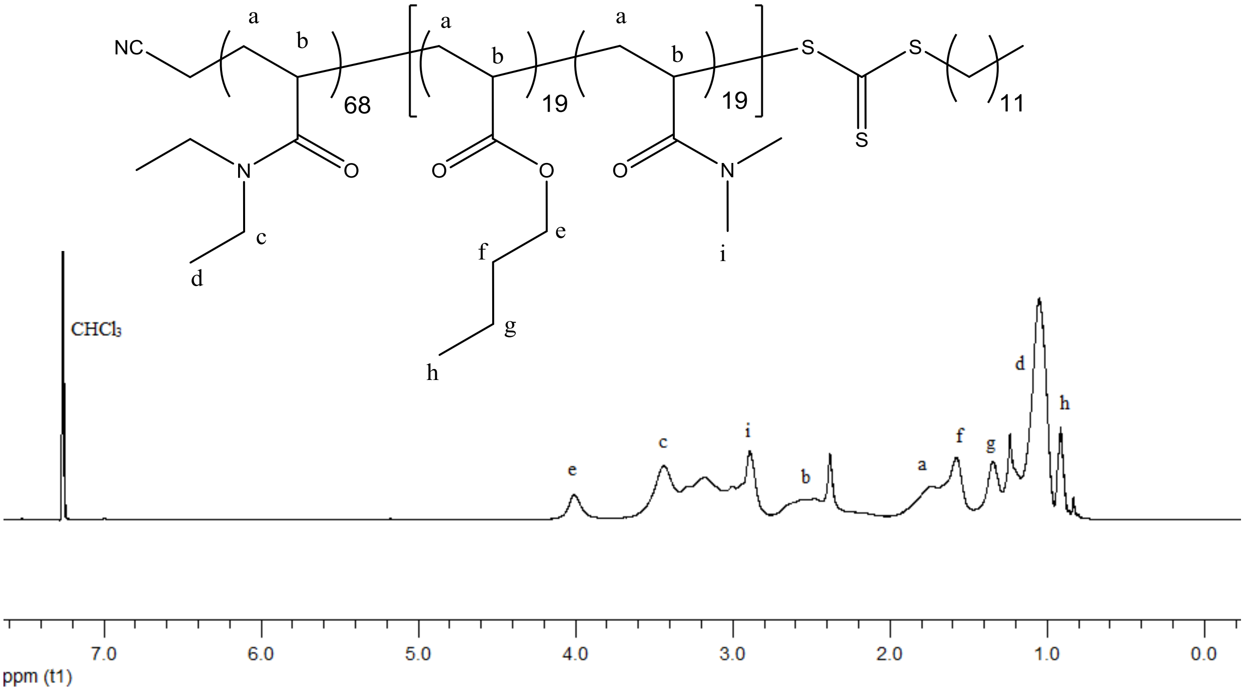

Figure S3. ^{1}H NMR spectrum of polymer **1**, analyzed at 400 MHz in $CDCl_3$.

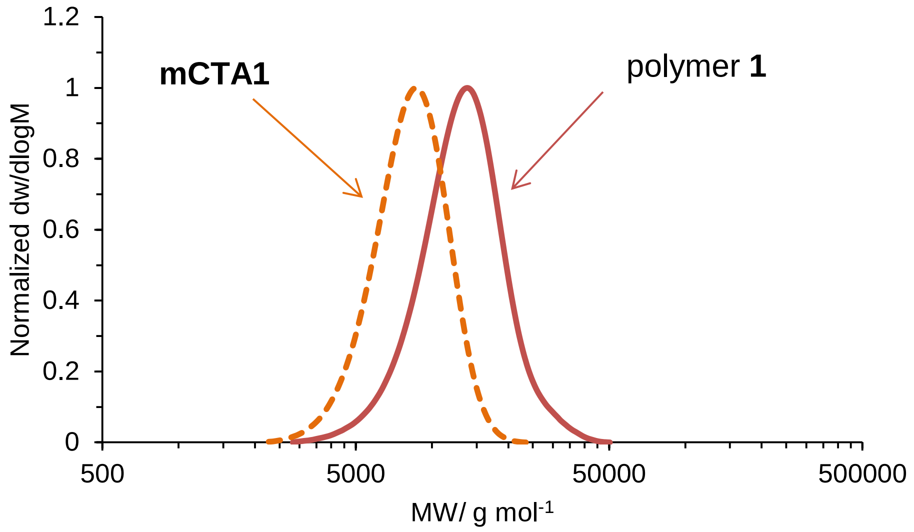

Figure S4. SEC molecular weight distributions of **mCTA1** and polymer **1**, using 2% TEA in THF as the eluent and calibrated against PMMA standards. In each case, the distributions were calculated using the RI traces.

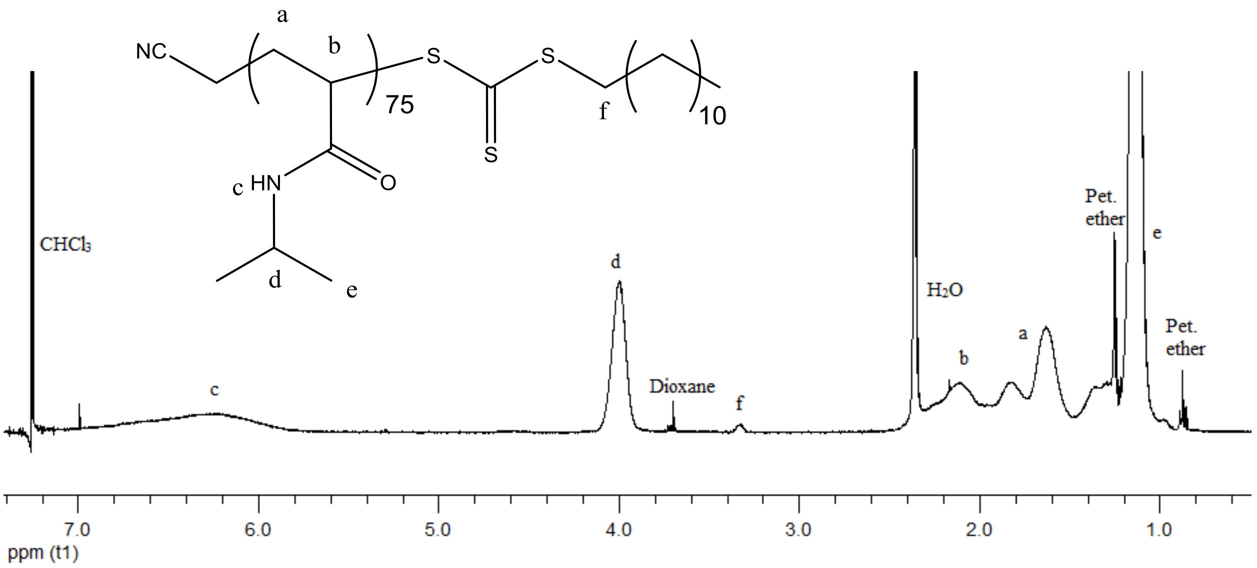

Figure S5. ^{1}H NMR spectrum of **mCTA2**, analyzed at 400 MHz in $CDCl_3$.

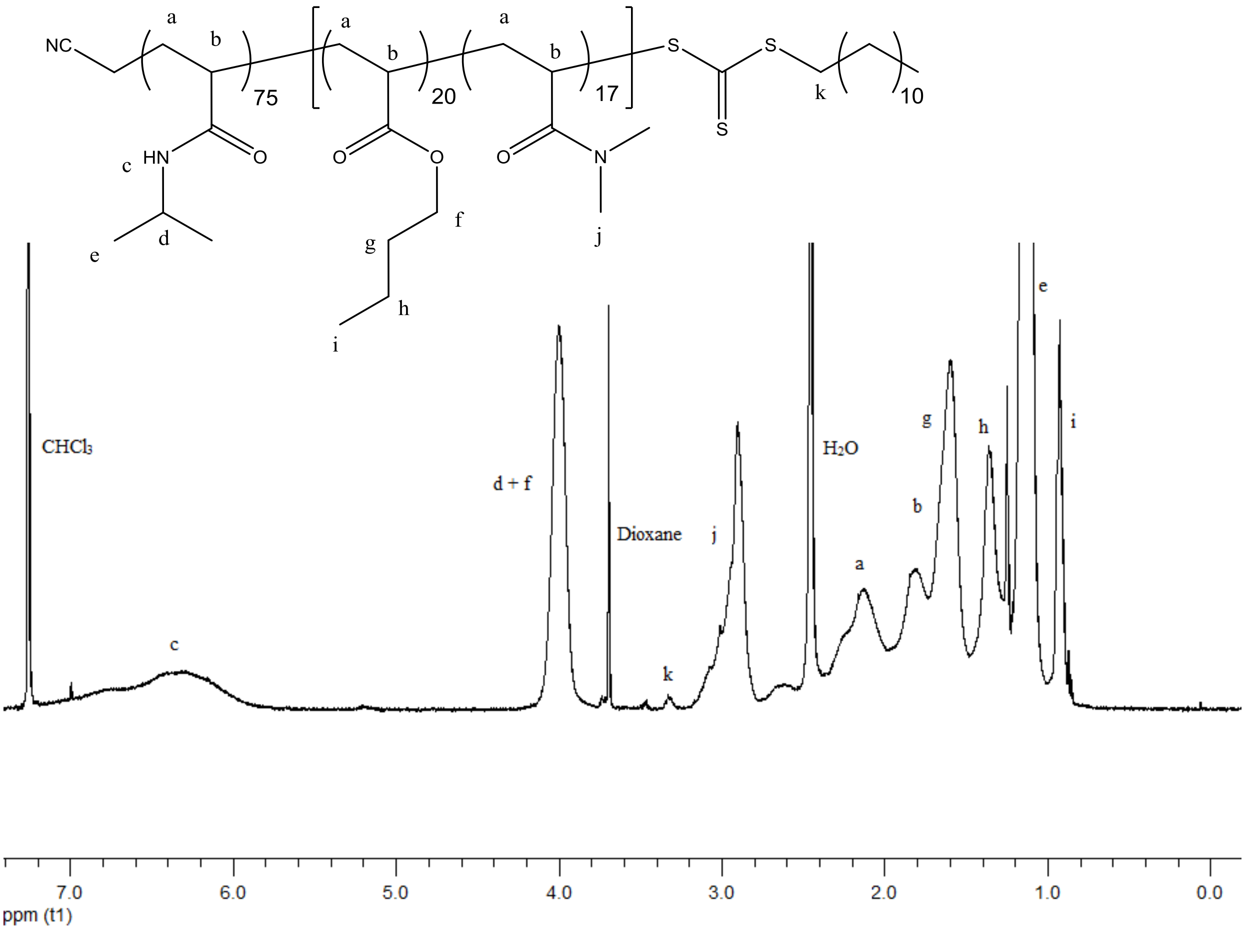

Figure S6. ^{1}H NMR spectrum of polymer **6**, analyzed at 400 MHz in $CDCl_3$.

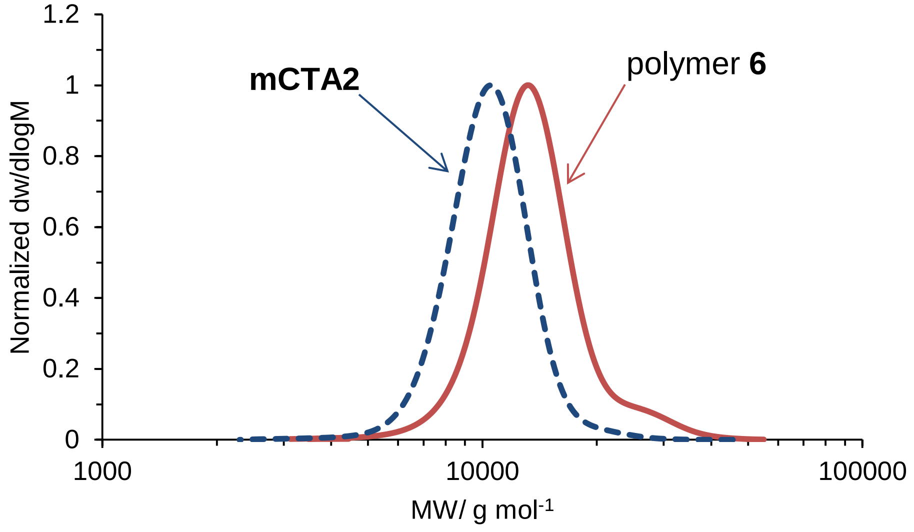

Figure S7. SEC RI chromatograms of **mCTA2** and polymer **6**, using 5 mM NH_4BF_4 in DMF as the eluent and calibrated against PMMA standards. In each case, the distributions were calculated using the RI traces.

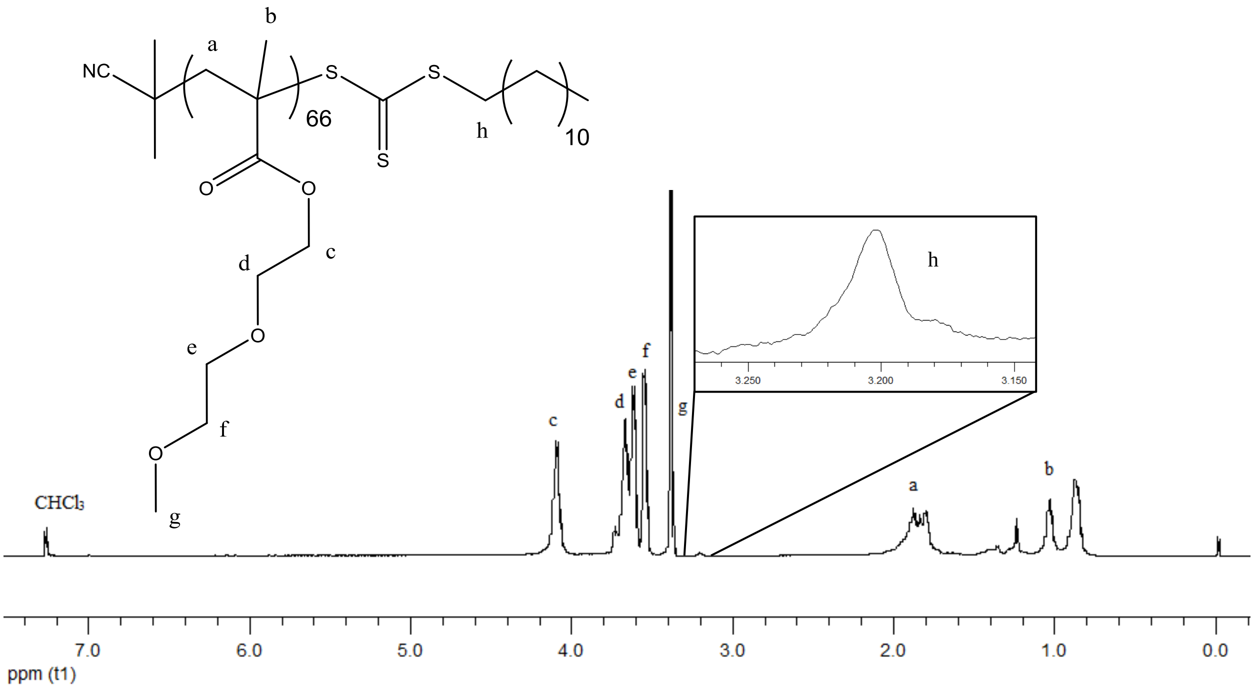

Figure S8. ^{1}H NMR spectrum of **mCTA3**, analyzed at 400 MHz in $CDCl_3$.

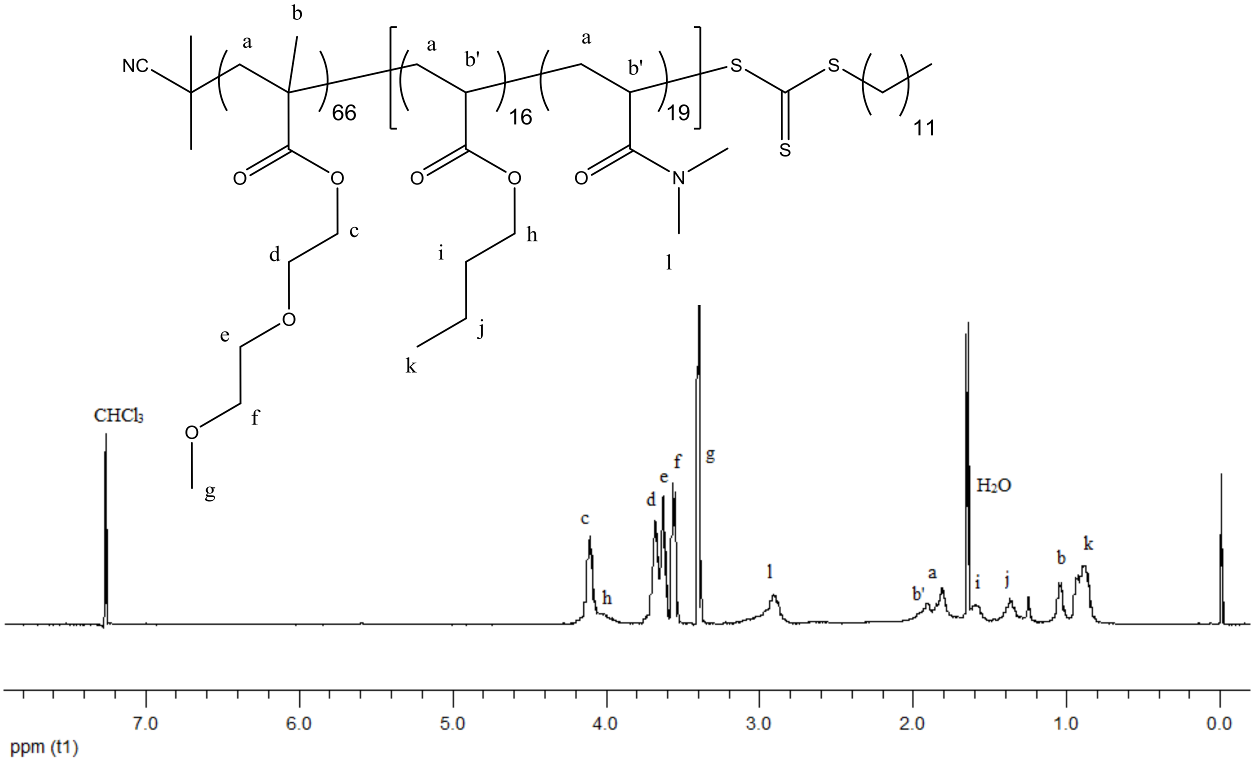

Figure S9. ^{1}H NMR spectrum of polymer **11**, analyzed at 400 MHz in $CDCl_3$.

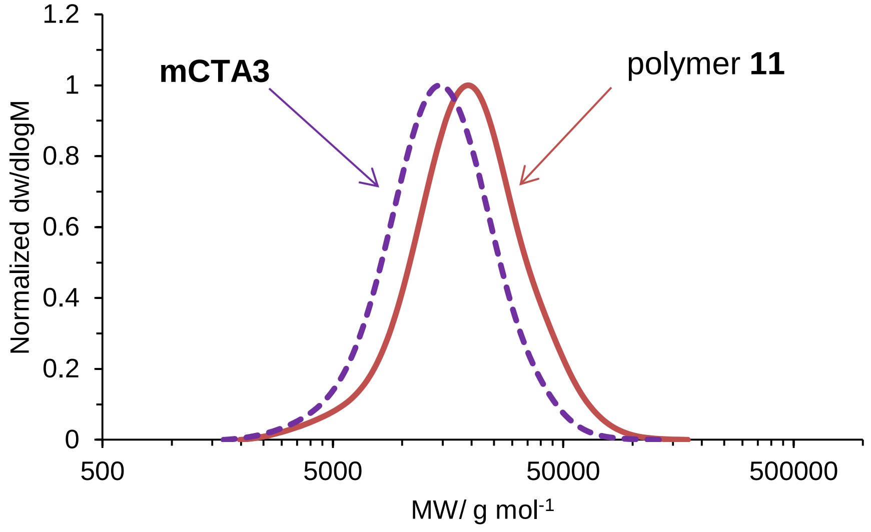

Figure S10. SEC RI chromatograms of **mCTA3** and polymer **11**, using 2% TEA in THF as the eluent and calibrated against PMMA standards. In each case, the distributions were calculated using the RI traces.

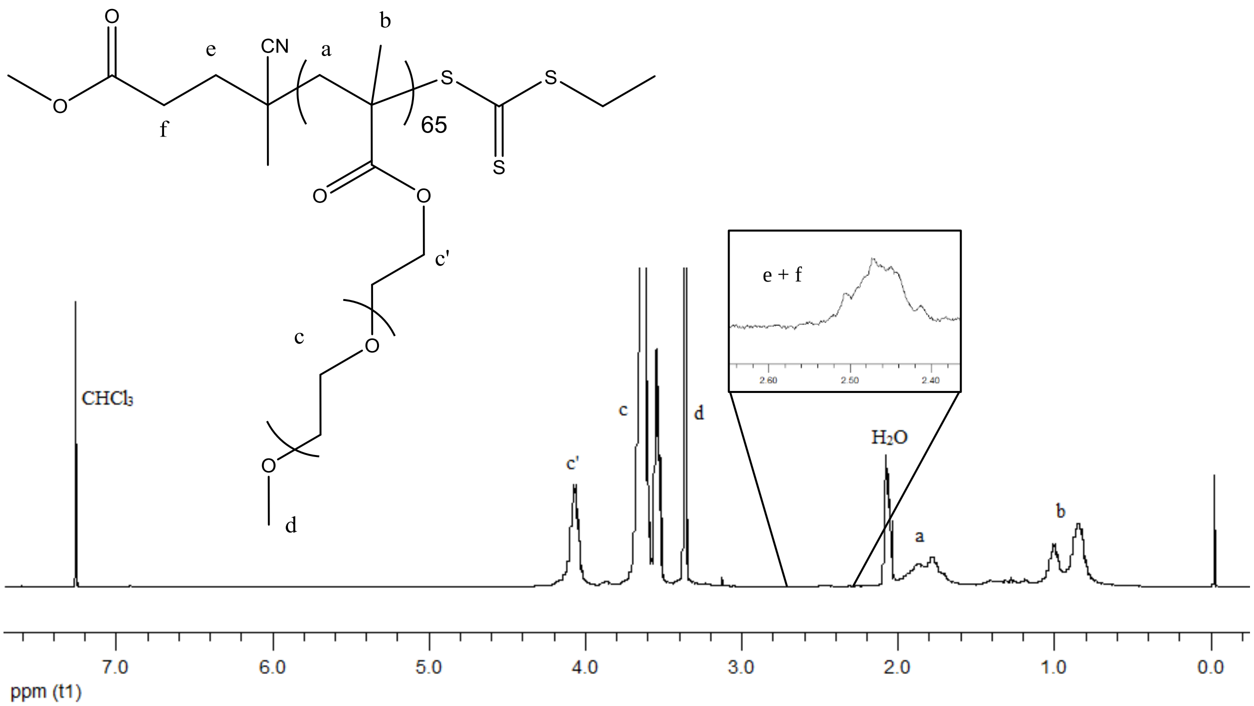

Figure S11. ^{1}H NMR spectrum of **mCTA4**, analyzed at 300 MHz in $CDCl_3$.

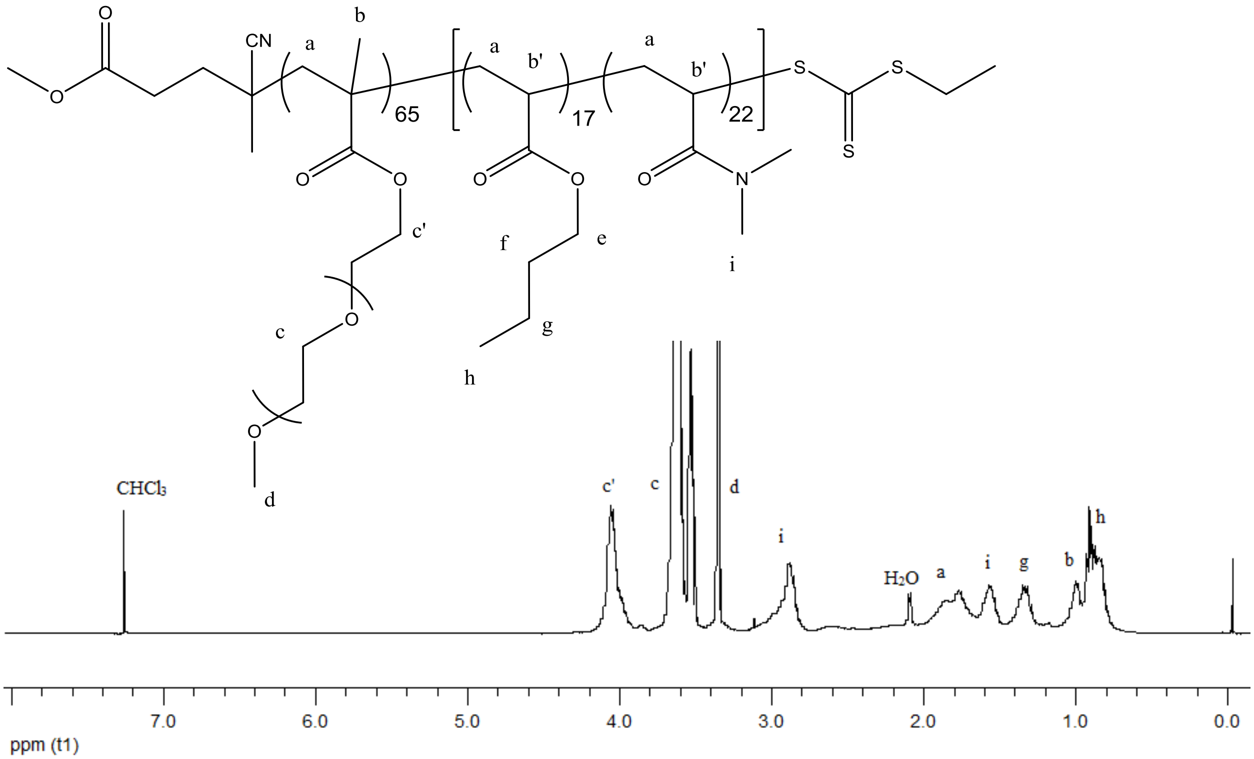

Figure S12. ^{1}H NMR spectrum of polymer **16**, analyzed at 300 MHz in $CDCl_3$.

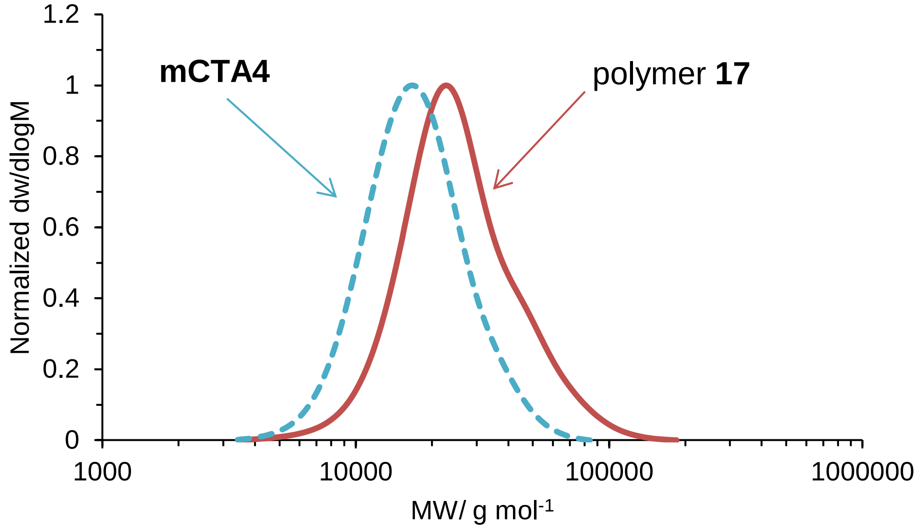

Figure S13. SEC RI chromatograms of **mCTA4** and polymer **17**, using 2% TEA in THF as the eluent and calibrated against PMMA standards. In each case, the distributions were calculated using the RI traces.

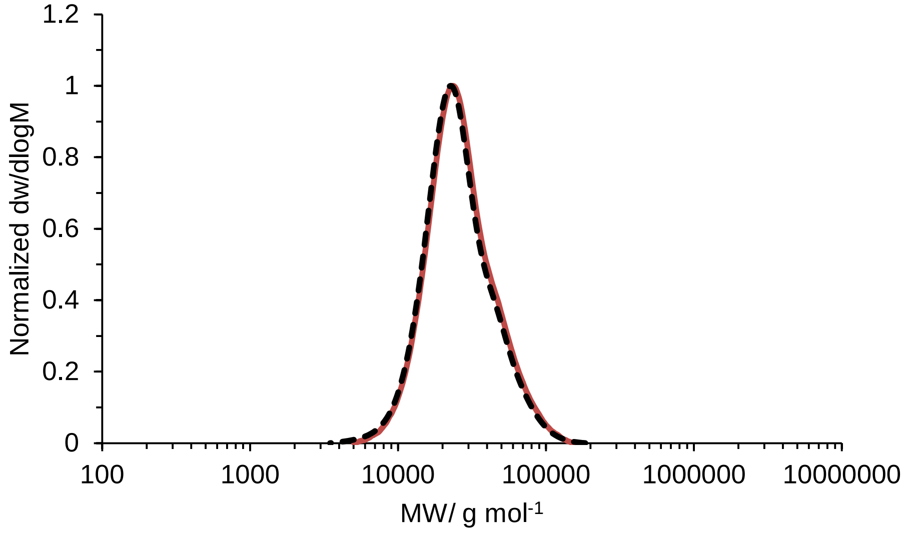

Figure S14. SEC RI chromatograms of polymer **17** before (black dashed line) and after (red solid line) three heating and cooling cycles from 50 - 95 °C. 2% TEA in THF was used as the eluent and the instrument was calibrated against PMMA standards. In each case, the distributions were calculated using the RI traces.

Representative light scattering data

DLS and SLS data for the 100 mol% *n*BA diblock copolymer micelles in each series is shown below

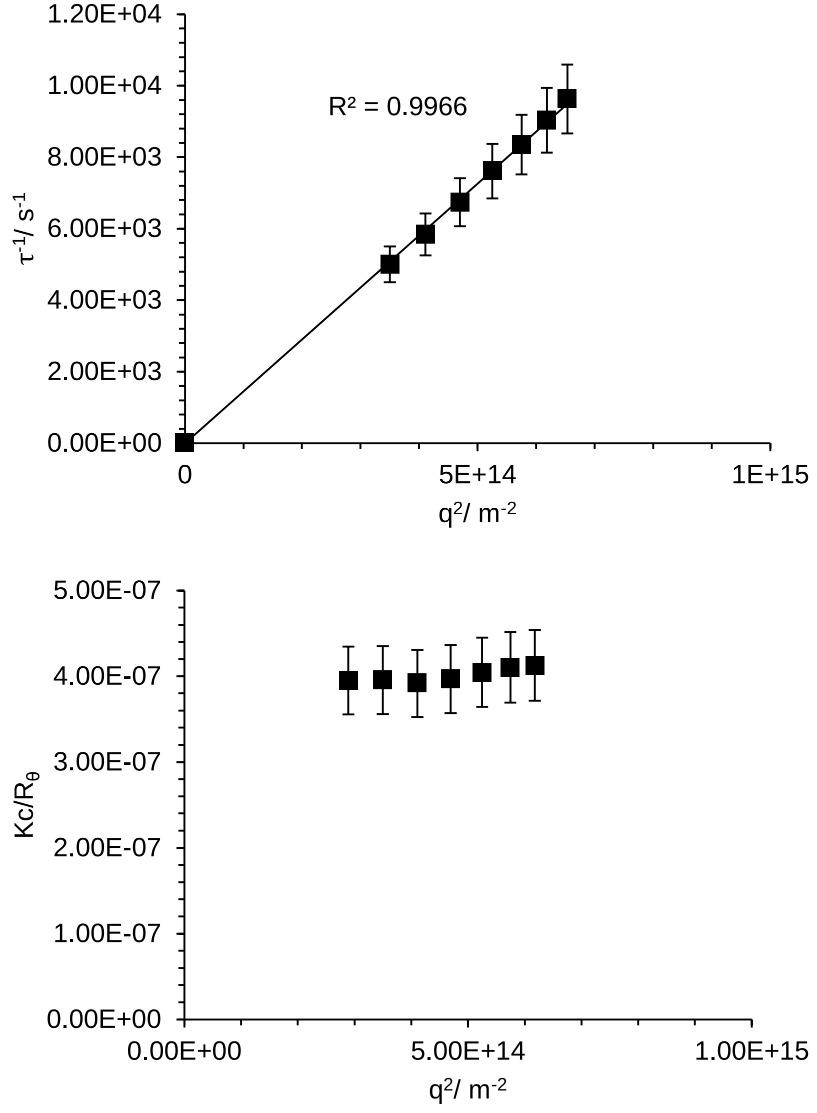

Figure S15. Multiple angle dynamic (above) and static (below) light scattering data of micelles comprised of polymer **5** at 1 mg mL^{-1}.

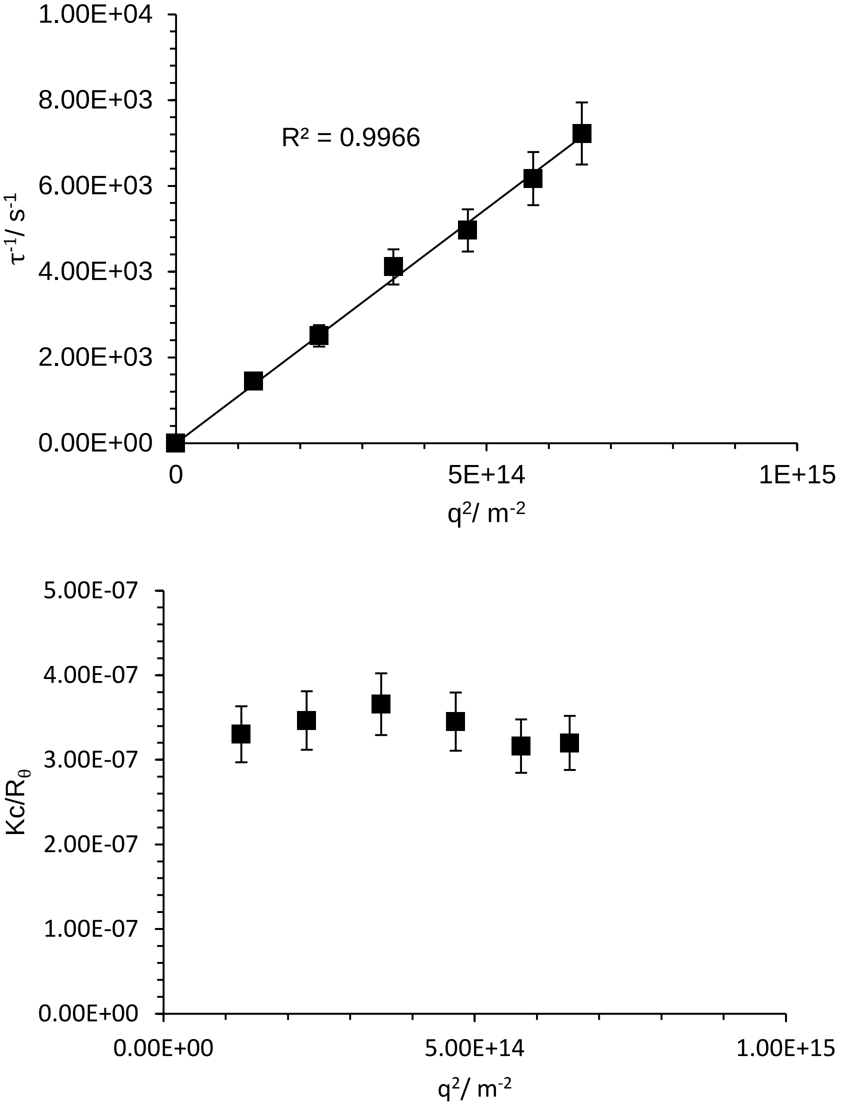

Figure S16. Multiple angle dynamic (above) and static (below) light scattering data of micelles comprised of polymer **10** at 1 mg mL^{-1}.

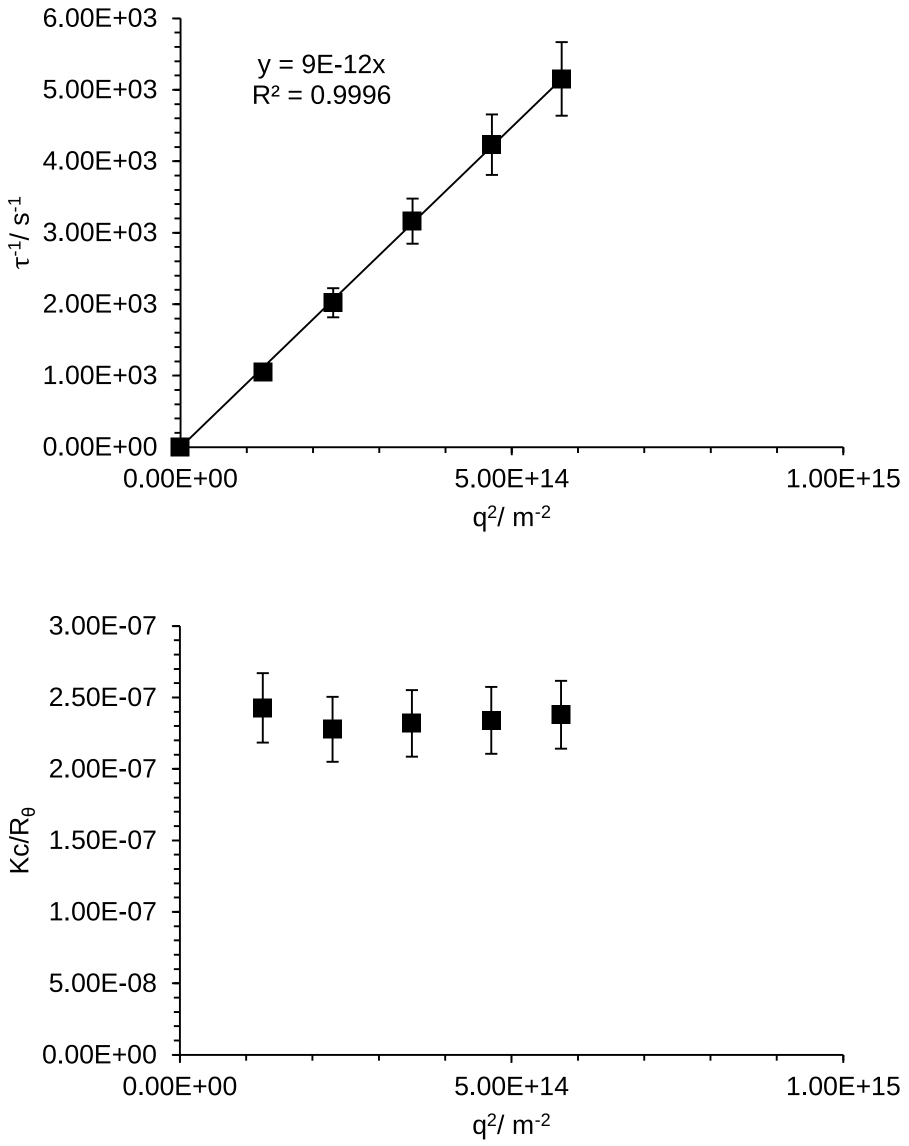

Figure S17. Multiple angle dynamic (above) and static (below) light scattering data of micelles comprised of polymer **15** at 1 mg mL^{-1}.

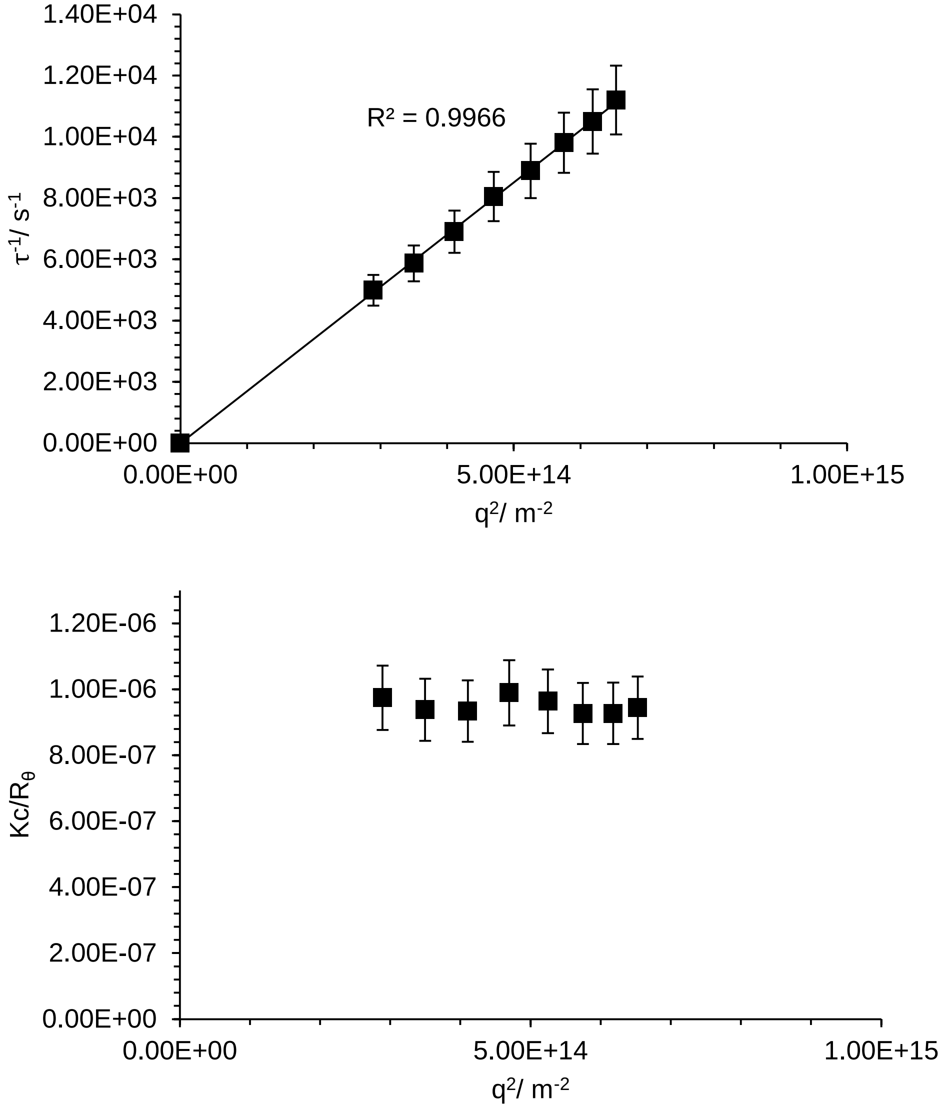

Figure S18. Multiple angle dynamic (above) and static (below) light scattering data of micelles comprised of polymer **17** at 1 mg mL^{-1}.

Representative turbidimetry data

Turbidimetry data for the 50 and 100 mol% *n*BA diblock copolymer micelles in each series is shown below.

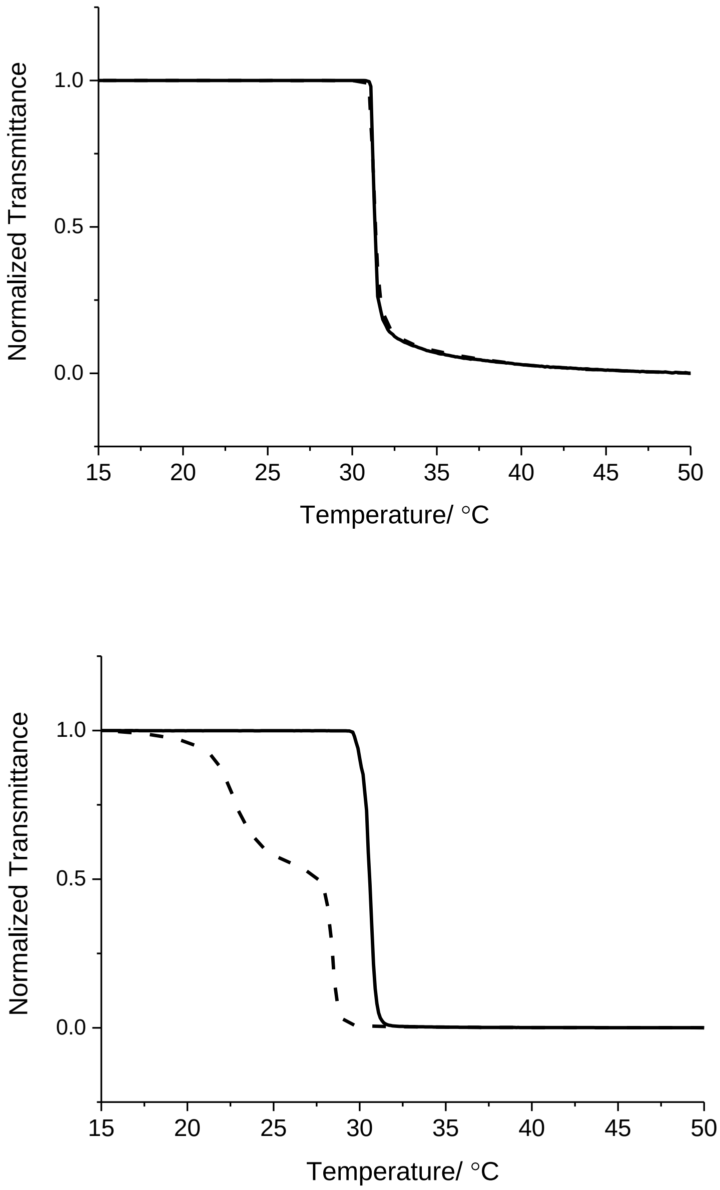

Figure S19. Variable temperature turbidimetry analysis of micelles comprised of polymers **1** (above) and **5** (below) at 1 mg mL^{-1}. In each case, the solid trace represents the heating cycle and the dashed trace represents the cooling cycle.

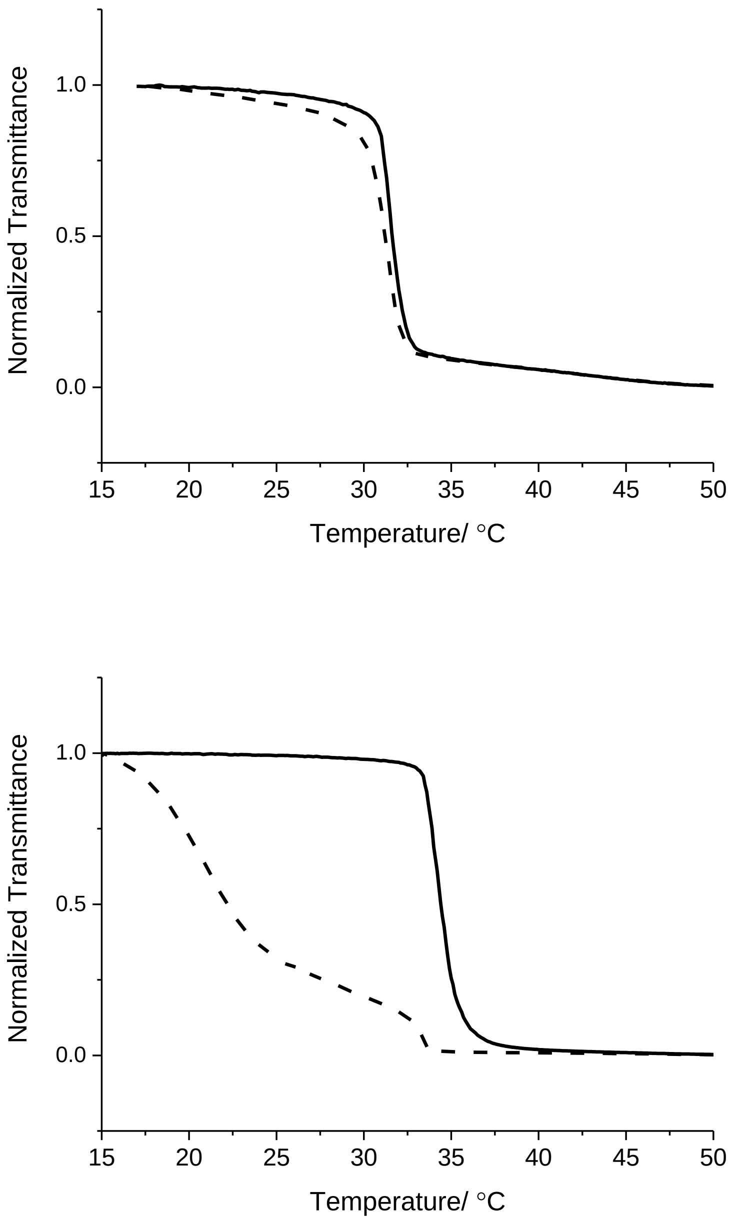

Figure S20. Variable temperature turbidimetry analysis of micelles comprised of polymers **6** (above) and **10** (below) at 1 mg mL^{-1}. In each case, the solid trace represents the heating cycle and the dashed trace represents the cooling cycle.

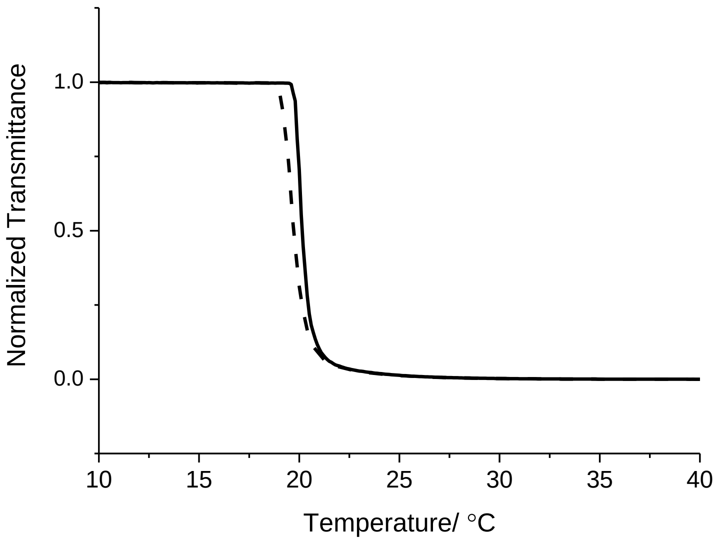

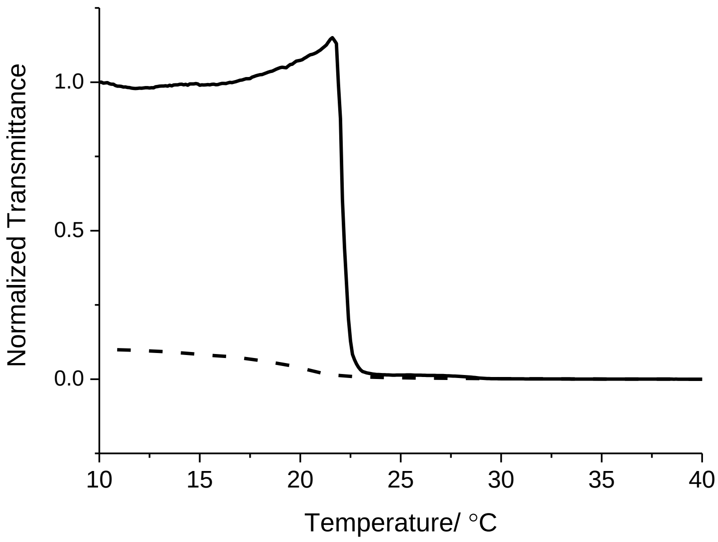

Figure S21. Variable temperature turbidimetry analysis of micelles comprised of polymers **11** (above) and **15** (below) at 1 mg mL^{-1}. In each case, the solid trace represents the heating cycle and the dashed trace represents the cooling cycle.

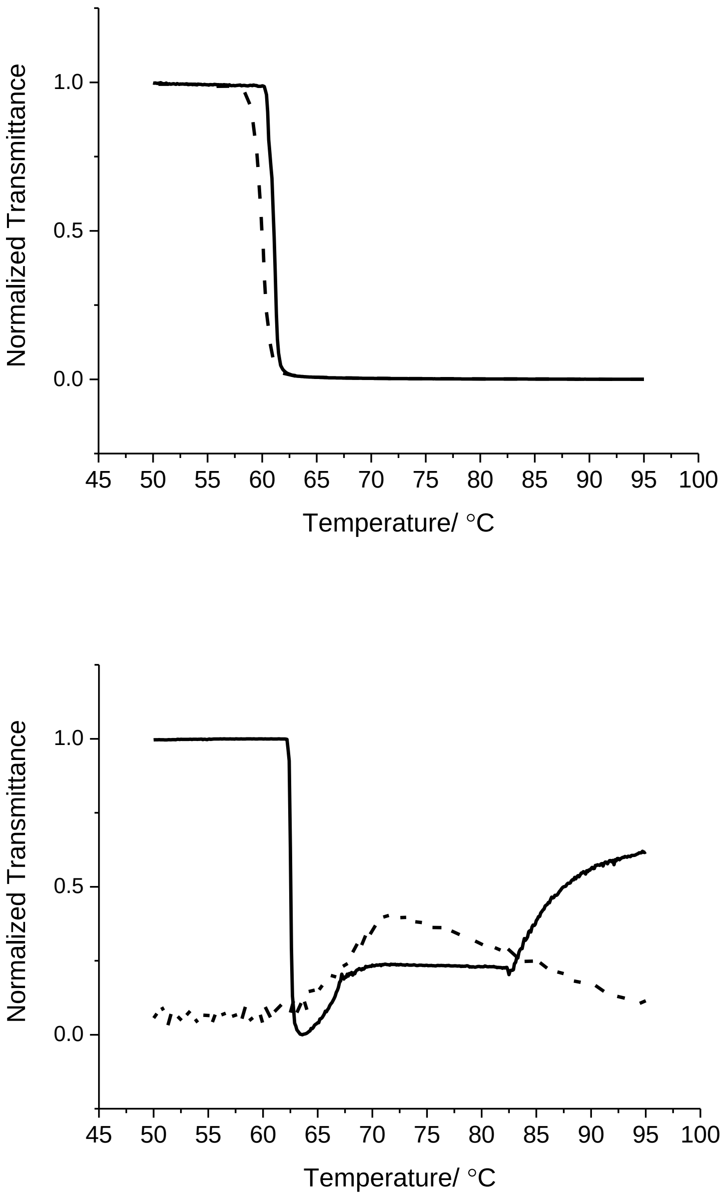

Figure S22. Variable temperature turbidimetry analysis of micelles comprised of polymers **16** (above) and **17** (below) at 1 mg mL^{-1}. In each case, the solid trace represents the heating cycle and the dashed trace represents the cooling cycle. For polymer **17** the trace of the cooling cycle has been smoothed for clarity. For polymer **17**, the rise in transmittance with temperature above 65 °C in the heating cycle, and subsequent variation in transmittance in the cooling cycle, was caused by sedimentation of the solid out of the path of the incident beam.

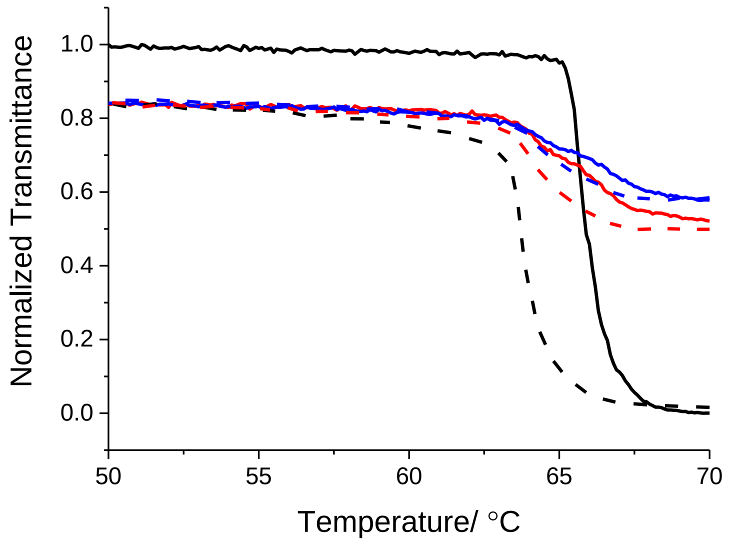

Figure S23. Variable temperature turbidimetry analysis of micelles comprised of polymer **17** at 1 mg mL^{-1} heated just past its transition temperature so as to reduce the thermal annealing time. In each case, the solid trace represents the heating cycle and the dashed trace represents the cooling cycle. Key: The 1st cycle (black traces), 2nd cycle (red traces) and 3rd cycle (blue traces) are shown.

Additional calculations and discussions

Calculation of core composition for polymers 1-5

Since the ^{1}H NMR peaks from **mCTA1** overlapped with the peaks from the p*n*BA-*co*-DMA core-forming block of polymers **1-5**, it was necessary to subtract the ^{1}H NMR spectrum of **mCTA1** from each of the spectra obtained for polymers **1-5**. This was achieved by normalizing the intensity of both spectra to a peak that remained unchanged by the chain extension, the signal at 3.44 ppm corresponding to the methylene protons in the macroCTA's side chain. Following subtraction, the integrals of the peaks at 4.00 and 3.22 - 2.77 ppm, corresponding to p*n*BA and pDMA respectively, were used to determine the core composition using the known DP of **mCTA1**.

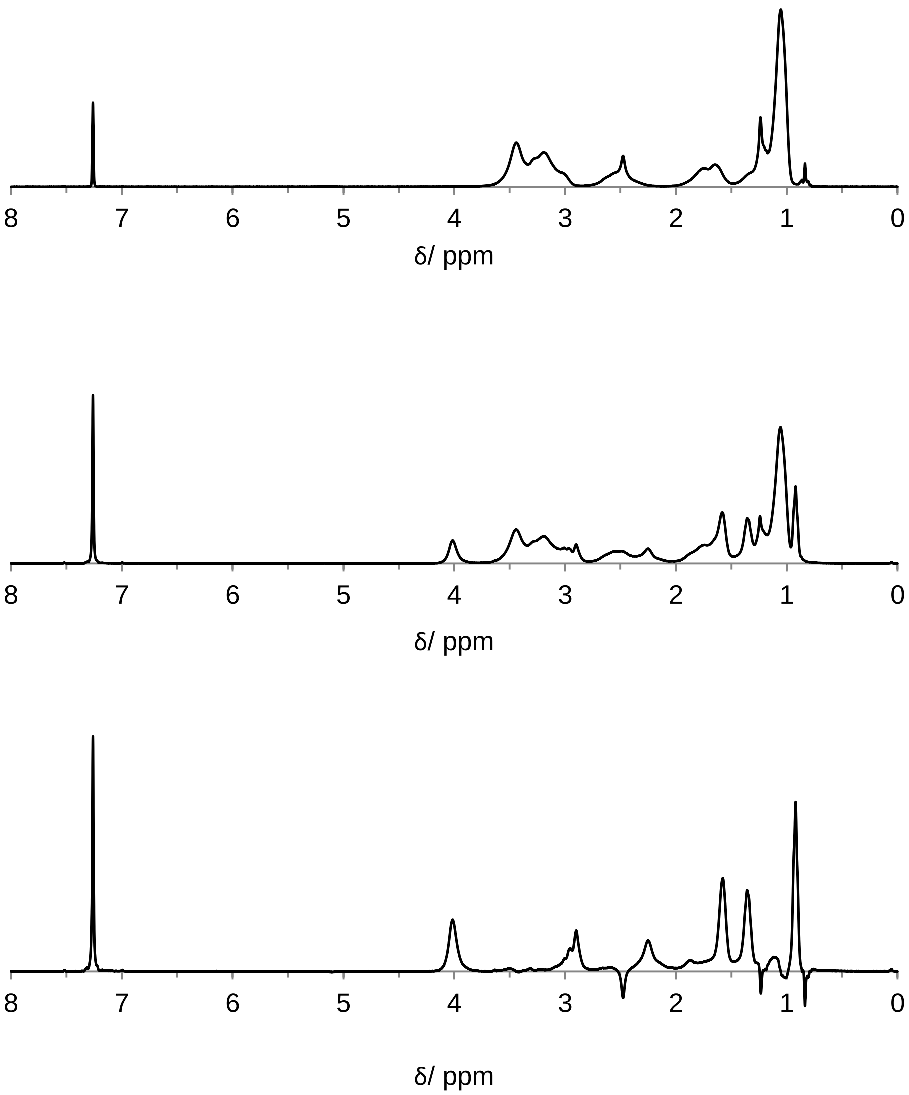

Figure S24. ^{1}H NMR spectra of **mCTA1** (top) and polymer **3** (middle). Below is the spectrum of **mCTA1** subtracted from that of polymer **3** used to calculate the core composition; the peaks at 4.00 and 3.22 - 2.77 ppm are clearly resolved.

Definitions and calculations regarding the light scattering data

The scattering wave vector, q, is defined in equation **(1)** where n is the refractive index of the solvent, λ is the wavelength of the incident beam and θ is the angle of measurement.

$$q = \frac{4\pi n}{\lambda}\sin\left(\frac{\theta}{2}\right) \quad \textbf{(1)}$$

The constrast factor, K, is defined in equation **(2)** where n_{standard} is the refractive index of the toluene standard, dn/dc is the refractive index increment of the sample, N_A is Avogadro's number and λ is the wavelength of the incident beam.

$$K = \frac{4\pi^2 n_{\text{standard}}{}^2\left(\frac{dn}{dc}\right)^2}{N_A\lambda^4} \quad \textbf{(2)}$$

The Rayleigh ratio of the sample, R_θ, is defined in equation **(3)** where I_{sample}, I_{solvent} and I_{standard} are the intensity of scattered light of the sample, solvent and standard respectively, detected for each angle of interest and $R_{\theta,\text{ standard}}$ is the Rayleigh ratio of the toluene standard.

$$R_\theta = \frac{I_{\text{sample}} - I_{\text{solvent}}}{I_{\text{standard}}} R_{\theta,\text{standard}} \quad \textbf{(3)}$$

R_{core} was calculated from N_{agg} using equation **(4)** where ρ is the composition-weighted density of the two monomers in the core-forming block and $M_{\text{w, core}}$ is the weight average molecular weight of the core-forming block, calculated by the number average molecular weight, M_n, determined by ^{1}H NMR multiplied by $Đ$ determined by SEC analysis.

$$\frac{4\pi\rho R_{\text{core}}{}^3}{3} = N_{\text{agg}}\frac{M_{\text{w,core}}}{N_A} \quad \textbf{(4)}$$

The average chain density within micelles comprised of pDEGMA (**11-15**) were calculated using equation **(5)**

$$Chain\ density = \frac{3N_{\text{agg}}}{4\pi R_H{}^3} \quad \textbf{(5)}$$

Discussion of the effect of chain density on reversibility for pDEGMA micelles

The chain density of the micelles with pDEGMA coronas (**11-15**) as calculated using equation **(5)** are plotted as a function of core composition in fig. S25. There are two clear regions, which correspond to micelles whose transitions were reversible and irreversible respectively.

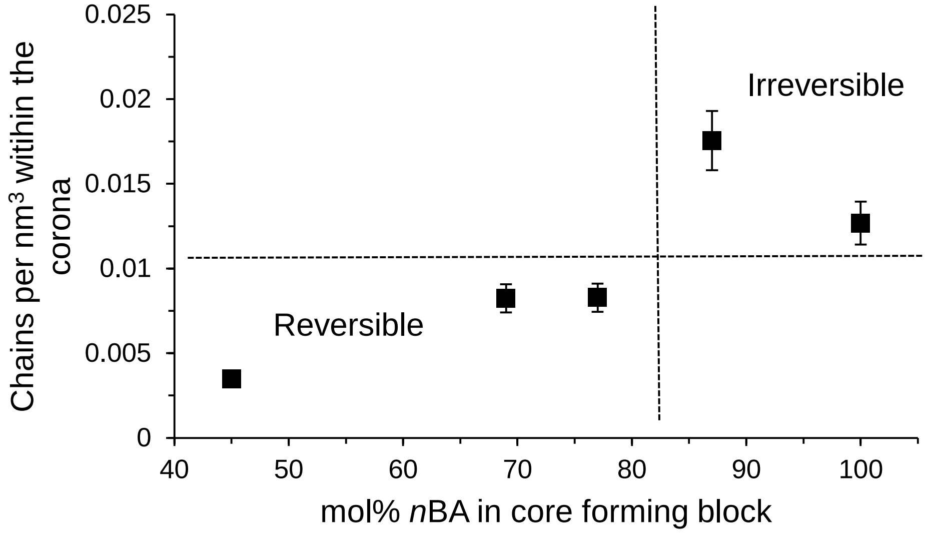

Figure S25. Chain density of micelles comprised of polymers **11-15**. Error bars represent 10% error. The two distinct regimes of reversible and irreversible phase transitions are marked with dashed lines. Note that polymer **14** (88% *n*BA in the core forming block) shows the highest chain density because its R_H is smaller than that of polymer **15** (100% *n*BA in the core forming block).
